# Particle Swarm Optimization for exploring Darcy–Forchheimer flow of Casson fluid between co-axial rotating disks with the Cattaneo–Christov model

**DOI:** 10.1038/s41598-024-56707-w

**Published:** 2024-04-03

**Authors:** Ziya Uddin, Himanshu Upreti, Sai Ganga, Wubshet Ibrahim

**Affiliations:** 1 SoET, BML Munjal University, Gurugram, Haryana India; 2 Department of Mathematics, Ambo University, Ambo, Ethiopia

**Keywords:** Casson fluid, Co-axial rotating disks, Temperature dependent thermal conductivity, Temperature dependent viscosity, Cattaneo–Christov heat flux model, Darcy–Forchheimer flow, Engineering, Mechanical engineering, Mathematics and computing, Applied mathematics, Computational science

## Abstract

In this paper, we carried out a numerical analysis of the fluid dynamics and heat transfer occurring between two parallel disks. The study accounts for the impact of temperature-dependent fluid viscosity and thermal conductivity. We systematically investigated various parameters, including viscosity, thermal conductivity, rotational behavior (rotation or counter-rotation), and the presence of stretching, aiming to comprehend their effects on fluid velocity, temperature profiles, and pressure distributions. Our research constructs a mathematical model that intricately couples fluid heat transfer and pressure distribution within the rotating system. To solve this model, we employed the 'Particle Swarm Optimization' method in tandem with the finite difference approach. The results are presented through visual representations of fluid flow profiles, temperature, and pressure distributions along the rotational axis. The findings revealed that the change in Casson factor from 2.5 to 1.5 resulted in a reduction of skin friction by up to 65%, while the change in local Nusselt number was minimal. Furthermore, both the viscosity variation parameter and thermal conductivity parameters were found to play significant roles in regulating both skin friction and local Nusselt number. These findings will have practical relevance to scientists and engineers working in fields related to heat management, such as those involved in rotating gas turbines, computer storage devices, medical equipment, space vehicles, and various other applications.

## Introduction

Rotating flow between co-axial disks holds great significance due to its ample applications such as in electric motors, cooling of rotating gas turbines, storage device in computers, medical equipment’s, space vehicle and others. The pioneering work in the direction of fluid flow over rotating disk was done by^1^. Later, this work was extended by^2^ for co-axial disk.

Stewartson^3^ investigated the flow of viscous fluid between a co-axial rotating disk. The findings were elucidated by theoretical and experimental models, and it was reported that "when the disk rotates in the opposite sense, the main body of the fluid is almost at rest." Holodniok et al.^4^ utilized the Newton–Raphson method to solve the equations governing the fluid flow between a co-axial rotating disk. Crewther et al.^5^ presented a theoretical study discussing the flow of viscoelastic fluid flowing between rotating parallel disks; both non-axisymmetric and axis-symmetric cases were discussed. Rajagopal^6^ conducted a graphical study to inspect the flow attributes of magnetized third-grade fluid. The flow was assumed to be unsteady, and the effect of the slip mechanism was considered. Asghar et al.^7^ investigated MHD non-Newtonian flows induced by non-coaxial rotations of an accelerated disk and a fluid at infinity. The study explored the complex interplay of rotational effects and magnetic fields, providing insights into the behavior of non-Newtonian fluids under such conditions. Ghosh et al.^8^ delved into the impact of Hall effects on MHD flows within a rotating system, the study addressed the coupling of magnetic fields with rotational effects. Aus der Wiesche^9^ contributed to the understanding of heat transfer phenomena in rotating flows. Ahmed et al.^10^ studied MHD swirling flows and heat transfer in a Maxwell fluid that was moved by two disks rotating in the same direction and having different thermal conductivities. Upadhya et al.^11^ investigated the nanofluid (graphene-ethylene glycol) flow between co-axial rotating disks. The model momentum equations are developed using quadratic Boussinesq approximation, with the assumption that one of the disks is porous and the other is non-porous. Nayak et al.^12^ focused on the interfacial layer and shape effects of the nanoparticles of CNTs dispersed in water to explore the flow features of electromagnetic Darcy–Forchheimer flow. The study reported that "augmenting the interfacial layer parameter enhances heat transportation from the surfaces of the lower and upper disks.” Flow instabilities under microgravity for fluid flowing between co-axial disk was investigated by Wang et al.^13^, Wang et al.^14^. Vijay and Sharma^15^ used FC-72-based nanofluid to examine the heat and mass transfer features of fluid flowing between co-axial rotating disks subjected to a magnetic field. Here, both disks are stretching radially, the viscosity of the nanofluid is treated as a function of temperature, and solutions are attained using HAM. Hussain and Xu^16^ examined the flow characteristics of micro-polar fluid. The fluid flow was confined between parallel disks subject to squeezing. The fluid flow was modeled utilizing the Buongiorno model and microorganism theory. The study reports that fluid temperature reduces with an enhancement in the suction parameter. Mehdi et al.^17^ applied HAM to unravel the solutions to the equations describing the magnetized flow of nanofluid flowing between co-axial disks of infinite length; the upper disk has a constant temperature, whereas the lower disk has an insulated surface.

Generally, the fluids which have practical relevance such as biological fluids, motor oils, polymeric fluids, paints and others are non-Newtonian fluids. As, these fluids have complex nature so it is not feasible to describe their characteristics by a single parent equation; hence different models have been given by researchers to explain their characteristics^18,19^. Casson fluid is one of the non-Newtonian fluids, defined as “Shear thinning liquid which is assumed to have an infinite viscosity at zero rate of shear, a yield stress below which no flow occurs, and zero viscosity at an infinite rate of shear”^20^. Tomato sauce, jelly, soup, honey, human blood are some fluids which exhibit their behavior as per Casson fluid. The practical significance of the non-Newtonian fluids and co-axial disk system have fascinated the researchers around the globe to analyze the flow characteristics using both. Researchers^21–24^ have extensively explored this complex field, methodically analyzing the behavior of Casson fluids under different flow situations.

Mohyud-Din and Khan^25^ examined the squeezing flow of Casson fluid between parallel disks. The heat transfer is due to the dissipation and thermal radiation; and the solution to the problem was obtained using HAM. Rafiq et al.^26^ considered the flow of Casson fluid between two porous co-axial rotating disks; the flow was assumed to be unsteady with disks being in two and fro motion and both upper and lower disk are subject to suction/injection effect. Hayat et al.^27^ examined the flow characteristics of Casson fluid flowing between rotating disks; the flow was assumed to be mixed convective and the system was exposed to physical process like chemical reaction, thermal radiation, convective heating and uniform source/sink of heat. Ramesh et al.^28^ examined the entropy generation of unsteady and mixed convective flow Casson fluid between parallel disks. The solution to the mathematical model was obtained using 4th order Runge–Kutta method; study reports that entropy increase with augmentation in the dissipation parameter. Liu et al.^29^ discussed the effects of binary chemical reaction, activation energy and convective mass transfer on flow characteristics (velocity, temperature and fractional mass transfer) of Casson fluid flowing between disks. The flow was assumed to be unsteady and medium was porous; the governing expressions were solved using bvp4c and implicit finite difference method. Abbas et al.^30^ examined the flow of Casson fluid between parallel disk, the flow motion was experienced due to the lower disk rotating continuously while the upper disk is stretching/shrinking radially. Noranuar et al.^31^ used CNTs based Casson nanofluid to inspect the heat and velocity field features of moving fluid between the rotating parallel disk. The flow medium was assumed to be porous and a uniform strength magnetic field was applied normal to the disks; and the solution to the governing equations are obtained using method of Laplace transform. Devaki et al.^32^ applied homotopy perturbation method to obtain the upshots to the equations describing the MHD flow of Casson fluid between co-axial disks. Jafar et al.^33^ examined the effect of thermal radiation, magnetic field and thermal stratification on flow characteristics (velocity, temperature and pressure) of Casson fluid flowing between two disks. The study deduced that temperature profile contracts with rise in the values of rotation and thermal stratification parameter. Akolade^34^ inspected the unsteady squeezing flow of Casson fluid passing through a channel of co-axial disk subject to the simultaneous effects of non-linear convection, magnetic field, chemical reaction, velocity slip and convective heat and mass transfer. The activation energy and the effects of the Casson-Maxwell nanofluid between fixed permeable discs were explored by^35^.

Industrial systems like catalytic reactors, fibrous insulation, heat exchanger and many others entail the convective flow through porous media. The flow in such system is usually explained via Darcy’s law, but this law doesn’t account the effects of inertial drag, vorticity diffusion, tortuosity. Hence, this law has some extension model, in this work authors considered the Darcy–Forchheimer model^36^. Hayat et al.^37^ analyzed the effect of thermal radiation, joule heating and nanoparticles volume fraction on heat and velocity profiles of nanofluid flowing between co-axial disks. The medium of the fluid is considered to be porous and flow through the channel was explicate using Darcy–Forchheimer model. The simulation of the MHD flow of nanofluid flowing through rotating parallel disk channel was done by Khan and Alzahrani^38^. A mathematical model deliberating the squeezing flow of nanofluid amid the two-coaxial disk rotating in their axis was presented by Riasat et al.^39^. This study accounts for the effects of magnetic field, partial slip, chemical reaction and nanoparticles concentration; and the fluid flowing in the space was modelled using Darcy–Forchheimer model. Other contribution in this direction can be accessed from^40,41^. Recently, Shahzad et al.^42^ examined Darcy–Forchheimer influences in a nanofluid flow. The research was conducted for the flow between two stretching discs. Radiation impacts and the significance of bioconvective factors were investigated. Basit et al.^43^ used the Darcy–Forchheimer model to study hybrid nanofluids between two spinning discs. Khan et al.^44^ proposed a mathematical model to analyze the entropy generation in viscous magnetized nanofluid flowing between parallel disks subject to radial stretching and axial rotation.

Dynamics of heat transfer have plethora of applications in industrial and bio-medical sectors. The mechanism of heat transfer is given by Fourier’s law based on continuum mechanics. This law has a major drawback, it does not account the time lag factor in heat transfer which makes this model quite unrealistic. Hence, several years later researchers,^45–49^ extended this law by including the time-lag factor and named it as Cattaneo–Christov heat flux model (CC model). Presently, much interest has been shown in the study of Cattaneo–Christov heat flux model, the investigations referenced from^50,51^ are few of the notable contributions examining the flow characteristics of nanofluid between co-axial disk using CC model. Nanofluid modelling is among the most recent Cattaneo Christov model applications. Titanium dioxide was used for the analysis by Zeb et al.^52^. Noreen et al.^53^ investigated the effects of hybrid nanofluids on double rotating discs under the impact of different factors using the Cattaneo Christov model.

The literature review reveals extensive research on heat transfer and fluid flow between parallel rotating disks. However, there is an unexplored dimension involving the application of the Cattaneo–Christov heat flux model in co-axial disk systems, particularly considering temperature-dependent fluid properties and non-linear convections. Furthermore, scant attention has been given to the examination of pressure distributions within such rotating systems. In light of these gaps, the present study is dedicated to analyzing these crucial factors, aiming to contribute valuable insights to the understanding of heat transfer and fluid dynamics in co-axial disk configurations under high temperature conditions.

## Problem statement and formulation

Consider two disks each with radii $r$ occupy the planes $z = 0$ and $z = h$ and are rotating with angular velocity $\Omega_{1}$ and $\Omega_{2}$ about *z*-axis in the same sense. A steady, incompressible and Darcy–Forchheimer flow of Casson fluid confined between the parallel disks. Both the disks are subject to radial stretching with stretching rates $A_{1}$ and $A_{2}$ and are assumed to be heating convectively. In this study, we considered that the flow model is subject to non-linear Boussinesq approximation (see 5th term on RHS of Eq. (3)). Owing to the practical utilization of non-linear Boussinesq approximation, the heating effect due to non-linear thermal radiation is accounted while heating due to porous medium is neglected. Here, the dynamic viscosity and thermal conductivity are assumed to be temperature dependent and other properties are independent of temperature.

**Figure 1:**
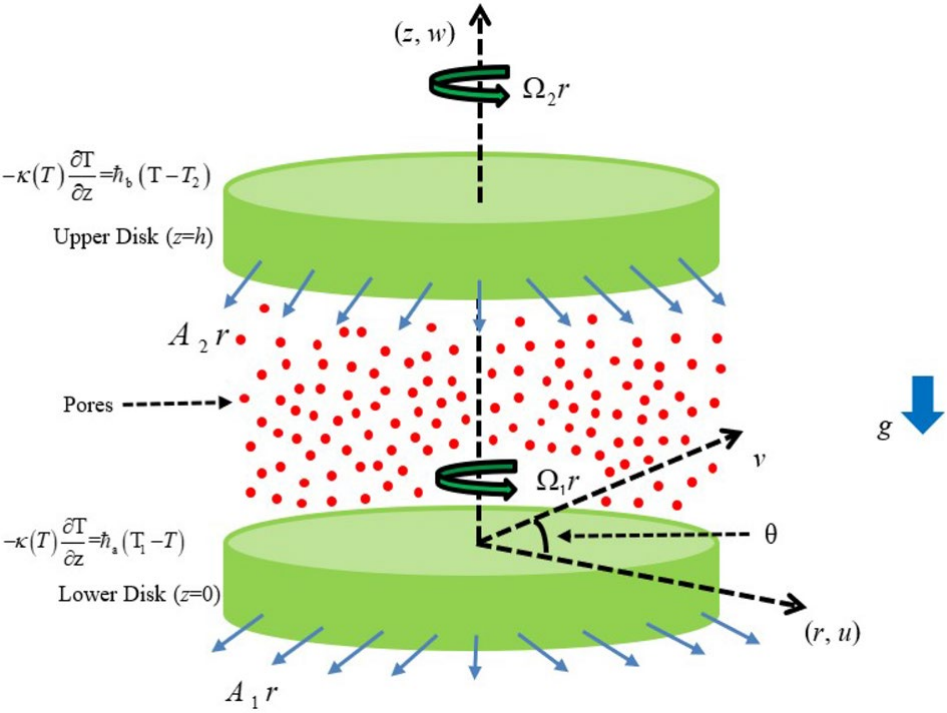
Schematic representation of the flow model.

The geometry illustrating the problem is shown in Fig. 1. The rheological equation which describes the relation of shear stress and strain rate for the incompressible flow of Casson fluid can be written as Upreti et al.^20^:

1 $$\tau_{ij} = \left\{ \begin{gathered} 2\left( {\mu_{B} + \left( {2\pi } \right)^{ - 1/2} P_{z} } \right)\varsigma_{ij,\;\;\;\;} \;\;\pi > \pi_{c} \hfill \\ 2\left( {\mu_{B} + \left( {2\pi_{c} } \right)^{ - 1/2} P_{z} } \right)\varsigma_{ij,\;\;\;\;} \;\pi < \pi_{c} \hfill \\ \end{gathered} \right.$$

In case of Casson fluid (non-Newtonian fluid), where $\pi > \pi_{c}$, it is possible to say that

$\mu = \mu_{B} + \left( {2\pi } \right)^{ - 1/2} P_{z} \;{\text{and}}\;\vartheta = \frac{{\mu_{B} }}{\rho }\left( {1 + \frac{1}{\delta }} \right)$ where $\delta = \mu_{B} \sqrt {2\pi } \left( {P_{z} } \right)^{ - 1}$ termed as Casson factor.

Here, symbols $\;\Gamma_{ij} ,\;\mu \;,\;\mu_{B} ,\;\vartheta ,\;P_{z} ,\;\pi \left( { = \varsigma_{ij\;\;} \varsigma_{ij\;\;} } \right),\;\pi_{c} \;{\text{and}}\;\varsigma_{ij\;\;}$ denotes Casson yield stress, absolute viscosity, plastic viscosity of non-Newtonian fluid, kinematic viscosity of Casson fluid, yield stress of the fluid, product of deformation rate, critical value of the product and component of deformation rate, respectively.

Under the above-stated assumptions along with the non-linear Boussinesq approximation^54^ and the Cattaneo-Christov heat flux model^49,53^, the current problem is governed as^11,25^:

2 $$\frac{\partial u}{{\partial r}} + \frac{\partial w}{{\partial z}} + \frac{u}{r} = 0$$

3 $$\begin{aligned} u\frac{\partial u}{{\partial r}} + w\frac{\partial u}{{\partial z}} - \frac{{v^{2} }}{r} & = - \frac{1}{\rho }\frac{\partial p}{{\partial r}} + \frac{1}{\rho }\left( {1 + \frac{1}{\delta }} \right)\left[ \begin{gathered} \frac{\partial }{\partial r}\left( {\mu_{B} \left( T \right)\frac{\partial u}{{\partial r}}} \right) + \frac{\partial }{\partial r}\left( {\mu_{B} \left( T \right)\frac{u}{r}} \right) \hfill \\ + \frac{\partial }{\partial z}\left( {\mu_{B} \left( T \right)\frac{\partial u}{{\partial z}}} \right) \hfill \\ \end{gathered} \right] \hfill \\ & \quad - \;\left( {1 + \frac{1}{\delta }} \right)\frac{{\mu_{B} \left( T \right)}}{\rho }\frac{u}{{k_{fH} }} - \;F_{o} u^{2} + g\left[ {\beta_{a} \left( {T - T_{2} } \right) + \beta_{b} \left( {T - T_{2} } \right)^{2} } \right] \hfill \\ \end{aligned}$$

4 $$\begin{aligned} u\frac{\partial v}{{\partial r}} + w\frac{\partial v}{{\partial z}} + \frac{uv}{r} & = \frac{1}{\rho }\left( {1 + \frac{1}{\delta }} \right)\left[ \begin{gathered} \frac{\partial }{\partial r}\left( {\mu_{B} \left( T \right)\frac{\partial v}{{\partial r}}} \right) + \frac{\partial }{\partial r}\left( {\mu_{B} \left( T \right)\frac{v}{r}} \right) \\ + \frac{\partial }{\partial z}\left( {\mu_{B} \left( T \right)\frac{\partial v}{{\partial z}}} \right) \hfill \\ \end{gathered} \right] \hfill \\& \quad - \left( {1 + \frac{1}{\delta }} \right)\frac{{\mu_{B} \left( T \right)}}{\rho }\frac{v}{{k_{fH} }} - F_{o} v^{2} \hfill \\ \end{aligned}$$

5 $$u\frac{\partial w}{{\partial r}} + w\frac{\partial w}{{\partial z}} = - \frac{1}{\rho }\frac{\partial p}{{\partial z}} + \frac{1}{\rho }\left( {1 + \frac{1}{\delta }} \right)\left[ \begin{gathered} \frac{\partial }{\partial r}\left( {\mu_{B} \left( T \right)\frac{\partial w}{{\partial r}}} \right) + r^{ - 1} \frac{\partial }{\partial r}\left( {\mu_{B} \left( T \right)w} \right) \hfill \\ + \frac{\partial }{\partial z}\left( {\mu_{B} \left( T \right)\frac{\partial w}{{\partial z}}} \right) \hfill \\ \end{gathered} \right]$$

6 $$\begin{gathered} u\frac{\partial T}{{\partial r}} + w\frac{\partial T}{{\partial z}} = \frac{1}{{\left( {\rho C_{p} } \right)}}\left[ {\frac{\partial }{\partial r}\left( {\kappa \left( T \right)\frac{\partial T}{{\partial r}}} \right) + \frac{1}{r}\frac{\partial }{\partial r}\left( {\kappa \left( T \right)T} \right) + \frac{\partial }{\partial z}\left( {\kappa \left( T \right)\frac{\partial T}{{\partial z}}} \right)} \right] \hfill \\ - \tau \left[ {u^{2} \frac{{\partial^{2} T}}{{\partial r^{2} }} + w^{2} \frac{{\partial^{2} T}}{{\partial z^{2} }} + 2uw\frac{{\partial^{2} T}}{\partial z\partial r} + \frac{\partial T}{{\partial r}}\left( {u\frac{\partial u}{{\partial r}} + w\frac{\partial u}{{\partial z}}} \right) + \frac{\partial T}{{\partial z}}\left( {u\frac{\partial w}{{\partial r}} + w\frac{\partial w}{{\partial z}}} \right)} \right] \hfill \\ + \frac{{16\sigma^{*} T^{3} }}{{3K^{*} \left( {\rho C_{p} } \right)}}\left[ {\frac{{\partial^{2} T}}{{\partial r^{2} }} + \frac{1}{r}\frac{\partial T}{{\partial r}} + \frac{{\partial^{2} T}}{{\partial z^{2} }}} \right] + \frac{{16\sigma^{*} T^{2} }}{{K^{*} \left( {\rho C_{p} } \right)}}\left[ {\left( {\frac{\partial T}{{\partial r}}} \right)^{2} + \left( {\frac{\partial T}{{\partial z}}} \right)^{2} } \right] \hfill \\ \end{gathered}$$

The flow is induced by the radial stretching or shrinking movement of the disks; also, the disks are supposed to be rotating along their axis of rotation. These result in centripetal and centrifugal forces, which in turn generate fluid flow. Additionally the disks undergo convective heating, therefore the respective boundary conditions are^44,49^:

7 $$\left. \begin{gathered} u = A_{1} r,{\text{ v = }}\Omega_{{1}} r,\;w = 0,\; - \kappa \left( T \right)\frac{{\partial {\text{T}}}}{{\partial {\text{z}}}}{ = }\hbar_{{\text{a}}} \left( {{\text{T}}_{{1}} - T} \right){\text{ at }}z = 0 \hfill \\ u = A_{2} r,{\text{ v = }}\Omega_{{2}} r,\;w = 0,\; - \kappa \left( T \right)\frac{{\partial {\text{T}}}}{{\partial {\text{z}}}}{ = }\hbar_{{\text{b}}} \left( {{\text{T}} - T_{2} } \right){\text{ at }}z = h \hfill \\ \end{gathered} \right\}$$

In above expressions, the symbols $u,\;v\;{\text{and}}\;w$ denotes velocity components $\left( {\text{m/s}} \right)$ in increasing direction of $r,\;\theta \;{\text{and}}\;z$,$\rho$ density $\left( {{\text{kgm}}^{{ - 3}} } \right)$ of working fluid , $C_{P}$ specific heat $\left( {\text{J/kgK}} \right)$, $k_{fH}$ Forchheimer permeability, $F_{o}$ inertial coefficient of porous medium, $g$ gravitational acceleration (m s^−2^), $\beta_{a} \;{\text{and}}\;\beta_{b}$ linear and non-linear thermal expansion coefficients $\left( {{\text{K}}^{{ - 1}} } \right)$, $p$ pressure $\left( {{\text{Pa}}} \right)$,$\tau$ heat flux relaxation time, $\sigma^{*}$ Stefan Boltzmann constant $\left( {{\text{Wm}}^{{2}} {\text{K}}^{{ - 4}} } \right)$, $K^{*}$ mean absorption coefficient $\left( {{\text{m}}^{ - 1} } \right)$, $\hbar_{a} \;{\text{and}}\,\hbar_{b}$ coefficients of convective heat transfer $\left( {{\text{Wm}}^{{2}} {\text{K}}^{{ - 1}} } \right)$, $T$ fluid temperature $\left( K \right)$ in the boundary layer.

Moreover, the dynamic viscosity $\left( {\mu_{B} \left( T \right)} \right)$ and thermal conductivity $\left( {\kappa \left( T \right)} \right)$ of the working fluid are considered being depending on temperature^10,55^:

8 $$\mu_{B} \left( T \right) = \frac{{\mu_{B}^{*} }}{{1 + \gamma \left( {T - T_{2} } \right)}}$$

9 $$\kappa \left( T \right) = \kappa_{\infty } \left[ {1 + \varepsilon \frac{{\left( {T - T_{2} } \right)}}{{\left( {T_{1} - T_{2} } \right)}}} \right]$$

Here, $\mu_{B}^{*} ,\;\gamma ,\;\;\kappa_{\infty } \;{\text{and}}\;\varepsilon$ refers to ambient fluid viscosity, non-negative constant, ambient fluid thermal conductivity and thermal conductivity parameter, respectively.

Now defining the following similarity transformation variables^10–12^

10 $$\left. \begin{aligned} \left( {u,\;v,\;w} \right) & = \left( {r\Omega_{1} F^{\prime}\left( \xi \right),\;r\Omega_{1} G\left( \xi \right),\; - 2h\Omega_{1} F\left( \xi \right)\;} \right), \hfill \\ \theta \left( \xi \right) & = \frac{{T - T_{2} }}{{T_{1} - T_{2} }},\;p = \rho_{f} \Omega_{1} \vartheta_{f} \left( {P\left( \xi \right) + \frac{{0.5r^{2} \varsigma }}{{h^{2} }}} \right),\;\xi = \frac{z}{h} \hfill \\ \end{aligned} \right\}$$

Here, $\varsigma$ is the pressure parameter and $\xi$ is the non-dimensional distance along the axis of rotation.

Making use of (8) and (9) and similarity variables (11), the continuity equations i.e., Eq. (2) is satisfied identically and the Eqs. (3)–(6) are transformed to following dimensionless form

11 $$\begin{gathered} \left( {1 + \frac{1}{\delta }} \right)\frac{1}{{\left[ {1 + E\left( {\theta \left( \xi \right)} \right)} \right]}}\left\{ {\frac{1}{{\text{Re} }}\left[ {F'''\left( \xi \right) - \frac{E}{{\left[ {1 + E\left( {\theta \left( \xi \right)} \right)} \right]}}F''\left( \xi \right)\theta '\left( \xi \right)} \right] - \bar{\omega }F'\left( \xi \right)} \right\} \hfill \\ \;\;\;\;\;\;\;\;\;\;\;\;\;\;\;\;\;\;\;\;\;\;\;\;\;\;\;\;\;\;\;\;\;\;\;\;\;\;\;\;\;\;\;\; - \left[ {1 + {Fr} } \right]\left( {F'\left( \xi \right)} \right)^{2} + \lambda _{1} \left[ {\theta \left( \xi \right) + \lambda _{2} \left( {\theta \left( \xi \right)} \right)^{2} } \right] \hfill \\ \;\;\;\;\;\;\;\;\;\;\;\;\;\;\;\;\;\;\;\;\;\;\;\;\;\;\;\;\;\;\;\;\;\;\;\;\;\;\;\;\;\;\;\;\;\;\;\;\;\;\;\;\;\;\;\; + 2F\left( \xi \right)F''\left( \xi \right) + \left( {G\left( \xi \right)} \right)^{2} - \frac{1}{{\text{Re} }}\varsigma = 0 \hfill \\ \end{gathered}$$

12 $$\begin{gathered} \left( {1 + \frac{1}{\delta }} \right)\frac{1}{{\left[ {1 + E\left( {\theta \left( \xi \right)} \right)} \right]}}\left\{ {\frac{1}{Re}\left[ {G^{\prime\prime}\left( \xi \right) - \frac{E}{{\left[ {1 + E\left( {\theta \left( \xi \right)} \right)} \right]}}G^{\prime}\left( \xi \right)\theta ^{\prime}\left( \xi \right)} \right] - \varpi G\left( \xi \right)} \right\} \hfill \\ - {Fr} \left( {G\left( \xi \right)} \right)^{2} + 2\left( {F\left( \xi \right)G^{\prime}\left( \xi \right) - F^{\prime}\left( \xi \right)G\left( \xi \right)} \right) = 0 \hfill \\ \end{gathered}$$

13 $$\begin{gathered} P^{\prime}\left( \xi \right) - \left( {1 + \frac{1}{\delta }} \right)\frac{2}{{\left[ {1 + E\left( {\theta \left( \xi \right)} \right)} \right]}}\left\{ {\frac{E}{{\left[ {1 + E\left( {\theta \left( \xi \right)} \right)} \right]}}F^{\prime}\left( \xi \right)\theta ^{\prime}\left( \xi \right) - F^{\prime\prime}\left( \xi \right)} \right\} \hfill \\ + 4ReF\left( \xi \right)F^{\prime}\left( \xi \right) = 0 \hfill \\ \end{gathered}$$

14 $$\begin{gathered} \left\{ {\varepsilon \left( {\theta ^{\prime}\left( \xi \right)} \right)^{2} + \left[ {1 + \varepsilon \theta \left( \xi \right)} \right]\theta ^{\prime\prime}\left( \xi \right)} \right\} + 4\tau_{T} RePr\left[ \begin{gathered} \left( {F\left( \xi \right)} \right)^{2} \theta ^{\prime\prime}\left( \xi \right) + \hfill \\ F\left( \xi \right)F^{\prime}\left( \xi \right)\theta ^{\prime}\left( \xi \right) \hfill \\ \end{gathered} \right] \hfill \\ + Ra\left[ \begin{gathered} \left( {\theta \left( \xi \right)\theta_{r} + 1} \right)^{3} \theta ^{\prime\prime}\left( \xi \right) + \hfill \\ 3\left( {\theta \left( \xi \right)\theta_{r} + 1} \right)^{2} \theta_{r} \left( {\theta ^{\prime}\left( \xi \right)} \right)^{2} \hfill \\ \end{gathered} \right] + 2RePrF\left( \xi \right)\theta ^{\prime}\left( \xi \right) = 0 \hfill \\ \end{gathered}$$

and boundary conditions (7) transformed to following forms:

15 $$\left. \begin{gathered} {\text{at}}\;\xi = 0:\;\;F\left( \xi \right) = 0,\;F^{\prime}\left( \xi \right) = A_{a} ,\;G\left( \xi \right) = 1,\;\theta ^{\prime}\left( \xi \right) = - \frac{{Bi_{a} }}{{\left[ {1 + \varepsilon \theta \left( \xi \right)} \right]}}\left[ {1 - \theta \left( \xi \right)} \right] \hfill \\ {\text{at}}\;\xi = 1:F\left( \xi \right) = 0,\;F^{\prime}\left( \xi \right) = A_{b} ,\;G\left( \xi \right) = \Omega ,\;\theta ^{\prime}\left( \xi \right) = - \frac{{Bi_{b} }}{{\left[ {1 + \varepsilon \theta \left( \xi \right)} \right]}}\theta \left( \xi \right) \hfill \\ \end{gathered} \right\}$$

Here, the symbols $\varpi ,\;{Fr} ,\,Re,\;E,\;\lambda_{1} ,\lambda_{2} ,Ra,\;Pr,\;\tau_{T} ,\;\theta_{r} ,\;\left( {A_{a} ,\;A_{b} } \right),\,\;\Omega \;,\;{\text{and}}\;\left( {Bi_{a} ,\;Bi_{b} } \right)$ present in the above expressions are refers to porosity parameter, Forchheimer parameter, Reynolds number, viscosity variation parameter, thermal buoyancy parameter, non-linear convection parameter, radiation parameter, Prandtl number, thermal relaxation parameter, temperature difference parameter, stretching parameter for lower and upper disks, rotation parameter, Biot numbers for lower and upper disks, respectively and are defined as

16 $$\left. \begin{aligned} \overline{\omega } & = \frac{\vartheta }{{\Omega_{1} k_{fH} }},\;{Fr} = rF_{o} ,\,Re = \frac{{\Omega_{1} h^{2} }}{\vartheta },\;E = \gamma \left( {T_{1} - T_{2} } \right),\;\lambda_{1} = \frac{{\alpha_{1} g\left( {T_{1} - T_{2} } \right)}}{{r\left( {\Omega_{1} } \right)^{2} }},\lambda_{2} = \frac{{\alpha_{2} \left( {T_{1} - T_{2} } \right)}}{{\alpha_{1} }}, \hfill \\ Ra & = \frac{{16\sigma^{*} T_{2}^{3} }}{{3K^{*} \kappa_{\infty } }},\;Pr = \frac{{\vartheta \left( {\rho C_{P} } \right)}}{{\kappa_{\infty } }},\;\tau_{T} = \tau \Omega_{1} ,\;\theta_{r} = \frac{{T_{1} }}{{T_{2} }} - 1,\;A_{a} = \frac{{A_{1} }}{{\Omega_{1} }},\;A_{b} = \frac{{A_{2} }}{{\Omega_{1} }},\,\;\Omega = \frac{{\Omega_{2} }}{{\Omega_{1} }},\; \hfill \\ Bi_{a} & = \frac{{\hbar_{a} h}}{{\kappa_{\infty } }},\;Bi_{b} = \frac{{\hbar_{b} h}}{{\kappa_{\infty } }} \hfill \\ \end{aligned} \right\}$$

Now, eliminating the pressure parameter $\varsigma$ from Eq. (11), and for this differentiating Eq. (11) with respect to $\xi$, we get the following equation

17 $$\begin{gathered} \left( {1 + \frac{1}{\delta }} \right)\left[ \begin{gathered} \frac{1}{{\left[ {1 + E\left( {\theta \left( \xi \right)} \right)} \right]}}\left\{ {\frac{1}{{\text{Re} }}\left[ \begin{gathered} F^{{iv}} \left( \xi \right) - \frac{E}{{\left[ {1 + E\left( {\theta \left( \xi \right)} \right)} \right]}}\left\{ \begin{gathered} F'''\left( \xi \right)\theta '\left( \xi \right) + \hfill \\ F''\left( \xi \right)\theta ''\left( \xi \right) \hfill \\ \end{gathered} \right\} + \hfill \\ \frac{{E^{2} }}{{\left[ {1 + E\left( {\theta \left( \xi \right)} \right)} \right]^{2} }}F''\left( \xi \right)\left( {\theta '\left( \xi \right)} \right)^{2} \hfill \\ \end{gathered} \right] - \bar{\omega }F''\left( \xi \right)} \right\} \hfill \\ - \frac{E}{{\left[ {1 + E\left( {\theta \left( \xi \right)} \right)} \right]^{2} }}\left\{ {\frac{1}{{\text{Re} }}\left[ {F'''\left( \xi \right) - \frac{E}{{\left[ {1 + E\left( {\theta \left( \xi \right)} \right)} \right]}}F''\left( \xi \right)\theta '\left( \xi \right)} \right] - \bar{\omega }F'\left( \xi \right)} \right\}\theta '\left( \xi \right) \hfill \\ \end{gathered} \right] \hfill \\ \;\;\;\;\;\;\;\;\;\;\;\;\;\;\;\;\;\;\; + \lambda _{1} \left[ {1 + 2\lambda _{2} \theta \left( \xi \right)} \right]\theta '\left( \xi \right) + 2\left[ {F\left( \xi \right)F'''\left( \xi \right) - 2{Fr} F'\left( \xi \right)F''\left( \xi \right) + G\left( \xi \right)G'\left( \xi \right)} \right] = 0 \hfill \\ \end{gathered}$$

Now, making use of (11) and (15), the following form of pressure parameter is obtained:

18 $$\begin{gathered} \varsigma = \left( {1 + \frac{1}{\delta }} \right)\frac{1}{{\left[ {1 + E\left( {\theta \left( 0 \right)} \right)} \right]}}\left\{ {\left[ {F'''\left( 0 \right) - \frac{E}{{\left[ {1 + E\left( {\theta \left( 0 \right)} \right)} \right]}}F''\left( 0 \right)\theta '\left( 0 \right)} \right] - \bar{\omega }\text{Re} F'\left( 0 \right)} \right\} \hfill \\ \;\;\;\;\;\;\;\;\;\;\;\;\;\;\;\;\;\;\;\;\;\;\;\;\;\;\;\;\;\;\;\; - \text{Re} \left\{ {\left[ {1 + {Fr} } \right]\left( {F'\left( 0 \right)} \right)^{2} + \lambda _{1} \left[ {\theta \left( 0 \right) + \lambda _{2} \left( {\theta \left( 0 \right)} \right)^{2} } \right] + \left( {G\left( 0 \right)} \right)^{2} } \right\} \hfill \\ \end{gathered}$$

To calculate the pressure term, Eq. (13) undergo integration with respect to $\xi$ from the limits $0$ to $\xi$, under the assumption of $P\left( {\xi = 0} \right) = 0$ we have

19 $$P\left( \xi \right) = - 2\left\{ {Re\left( {F\left( \xi \right)} \right)^{2} + \left( {1 + \delta^{ - 1} } \right)\left[ {1 + E\left( {\theta \left( \xi \right)} \right)} \right]^{ - 1} \left[ {F^{\prime}\left( \xi \right) - F^{\prime}\left( 0 \right)} \right]} \right\}$$

The skin friction coefficient $\left( {Cf_{x} } \right)$ and local Nusselt number $\left( {Nu_{x} } \right)$ at lower $\left( {\xi = 0} \right)$ and upper $\left( {\xi = 1} \right)$ disks are given as Khan et al.^44^

20 $$\left. \begin{aligned} Cf_{xa} Re_{r} & = \frac{{1 + \delta^{ - 1} }}{{1 + E\theta \left( {\xi = 0} \right)}}\left[ {\left( {F^{\prime\prime}\left( {\xi = 0} \right)} \right)^{2} + \left( {G^{\prime}\left( {\xi = 0} \right)} \right)^{2} } \right]^{0.5} \hfill \\ Nu_{xa} & = - \left[ {\left( {1 + \varepsilon \theta \left( {\xi = 0} \right)} \right) + Ra\left[ {1 + \theta_{r} \theta \left( {\xi = 0} \right)} \right]^{3} } \right]\theta ^{\prime}\left( {\xi = 0} \right)\; \hfill \\ \end{aligned} \right\}$$

21 $$\left. \begin{aligned} Cf_{xb} Re_{r} & = \frac{{1 + \delta^{ - 1} }}{{1 + E\theta \left( {\xi = 1} \right)}}\left[ {\left( {F^{\prime\prime}\left( {\xi = 1} \right)} \right)^{2} + \left( {G^{\prime}\left( {\xi = 1} \right)} \right)^{2} } \right]^{0.5} \hfill \\ Nu_{xb} & = - \left[ {\left( {1 + \varepsilon \theta \left( {\xi = 1} \right)} \right) + Ra\left[ {1 + \theta_{r} \theta \left( {\xi = 1} \right)} \right]^{3} } \right]\theta ^{\prime}\left( {\xi = 1} \right) \hfill \\ \end{aligned} \right\}$$

where $Re_{r} = \frac{\vartheta }{{r\Omega_{1} h}}$ is local Reynolds number.

## Numerical methodology

The non-dimensional mathematical model, detailed in Eqs. (12), (14) and (17) along with the boundary conditions (15), has been effectively addressed through numerical methods. Initially, we restructured all equations into a system of eight first-order differential equations, adhering to the specified boundary conditions (Eq. 15). However, for solving this system using a finite difference scheme, all eight initial conditions are required. Unfortunately, the formulated problem only provides four initial conditions (Eq. 15). Hence, to resolve this system, we must compute the remaining four missing initial conditions using the four known boundary conditions, also delineated in Eq. (15). To achieve this, we employ the "Particle Swarm Optimization" method in conjunction with the Runge–Kutta-Fehlberg scheme. We establish an error function, termed the root of residuals (RR), which is minimized to obtain the missing boundary condition and hence the final solution of the problem. A comprehensive description of this methodology can be found in^20^.

In PSO the swarms are updated by using the following equations.

22 $$v\left[ {} \right] = \psi *v\left[ {} \right] + \phi_{p} *rand()*(Pbest\left[ {} \right] - x\left[ {} \right]) + \phi_{g} *rand()*(Gbest\left[ {} \right] - x\left[ {} \right])$$

23 $$x\left[ {} \right] = x\left[ {} \right] + v\left[ {} \right]$$

The overall objective of the proposed method is to determine the initial such that the following error function is minimized.

24 $$RR=\sqrt{{{\left\{F(1)\right\}}^{2}+{\left\{{F}^{\mathrm{^{\prime}}}\left(1\right)-{A}_{b}\right\}}^{2}+{\left\{G\left(1\right)-\Omega \right\}}^{2}+\left\{{\theta }^{\mathrm{^{\prime}}}\left(1\right)+\frac{{Bi}_{b}}{1+\varepsilon \theta \left(1\right)}\theta (1)\right\}}^{2}}$$

The process is implemented with the help of computer code written in MATLAB. The minimization of this “RR” is achieved with the help of PSO under the convergence criteria of $RR\le {10}^{-08}$. The values of PSO convergence controlling parameters are taken as ${\phi }_{p}={\phi }_{p}=1.49618$ and $\psi =0.7298$ .

The flow chart for the computational procedure is given below.
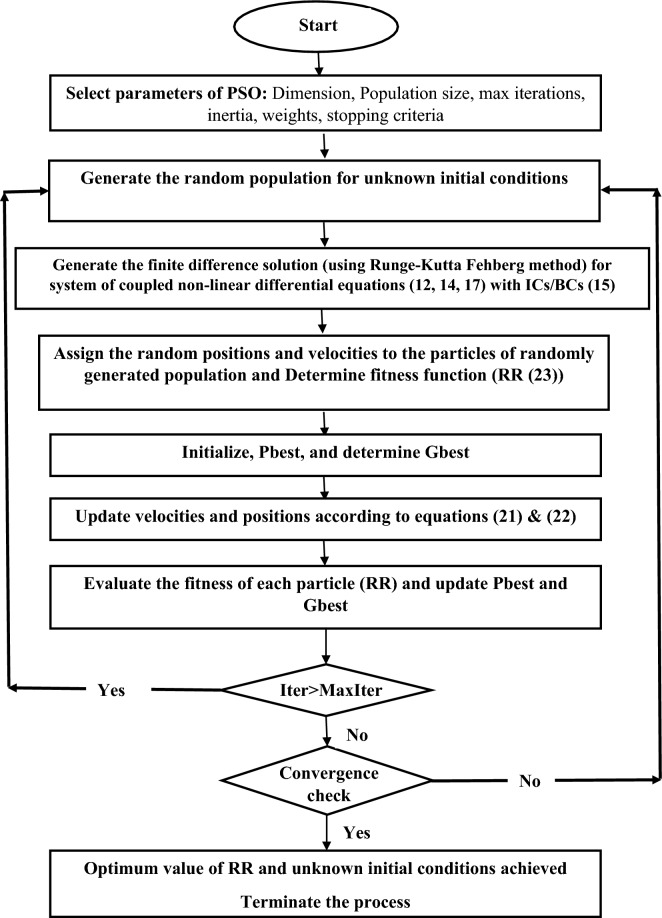


To validate the methodology, we solved a specific case of the presented problem and compared the results with those from previously published work. The comparison presented in Table 1 below serves to illustrate the credibility and validity of the proposed methodology.

**Table 1:** Comparison of results $\overline\omega { = 0,}\;Fr{ = 0,}\;Re = 1,\;E = 0,\;\varepsilon = 0,\;\lambda_{1} = 0 = \lambda_{2} ,$ $Pr = 6.2,\;\tau = 1,\;\theta_{r} = 0,$ and $A_{a} = 0 = A_{b}$.

$\Omega$	Present results	Turkyilmazoglu^56^	Present results	Turkyilmazoglu^56^
	$F^{\prime \prime}(0)$	$F^{\prime \prime}(0)$	$-G^{\prime}(0)$	$-G^{\prime}(0)$
− 1.0	0.0666	0.06666313	2.0001	2.00095215
− 0.8	0.0839	0.08394206	1.8026	1.80258847
− 0.3	0.1039	0.10395088	1.3044	1.30442355
0	0.0999	0.09997221	1.0043	1.00427756
0.5	0.0666	0.06663419	0.5026	0.50261351

## Results and discussion

The current section deals with the visualization of the influence of parameters like $A_{a} ,\;A_{b} ,\;Re,\;Bi_{a}$ and $\Omega$ on the fluid’s velocity (axial $F\left( \xi \right)$, radial $F^{\prime}\left( \xi \right)$ and tangential $G\left( \xi \right)$), temperature $\theta \left( \xi \right)$ and pressure $P\left( \xi \right)$; all the flow attribute profiles are plotted against distance along the axis of rotation $\left( \xi \right)$. In the computations, the acceptable range assigned to the parameters for better graphical results is as follows $- 1 \le A_{a} ,\;A_{b} ,\;\Omega \le 1,\;2 \le Re \le 7$, $0.1 \le Bi_{a} \le 0.5$ and other parameters are treated as constant quantities. From the graphs, it is clear that the obtained solutions satisfy both the initial and boundary conditions (15). And, the quantities like SFC and LNN are computed for the lower and upper discs for the aforesaid parameters and presented through Tables 2 and 3.

**Table 2:** Computed values of SFC and LNN when $\delta = 0.8,\;\overline\omega = 0.5,\;Fr = 1,\;Re = 2,\;E = 0.2 = \varepsilon ,$ $\lambda_{1} = 1 = \lambda_{2} ,$ $Ra = 0.5,\;\theta_{r} = 0.5,\;Bi_{a} = 0.4 = Bi_{b}$.

$\Omega$	$A_{a}$	$A_{b}$	Lower disk		Upper disk	
			SFC	LNN	SFC	LNN
− 1	− 1	0.5	6.9318	0.2107	4.7907	0.5019
	− 0.5		4.5865	0.2294	5.0829	0.4679
	0		4.7758	0.2687	6.1089	0.4080
	0.5		7.8149	0.3256	7.5481	0.3285
	1		12.058	0.3789	9.1723	0.2583
0	− 1		6.3884	0.2110	1.7490	0.5013
	− 0.5		3.4390	0.2301	2.9381	0.4666
	0		3.3465	0.2699	4.7511	0.4059
	0.5		6.8262	0.3274	6.6696	0.3259
	1		11.299	0.3805	8.5944	0.2560
1	− 1		5.9351	0.2107	2.0503	0.5019
	− 0.5		2.5014	0.2294	2.9612	0.4679
	0		2.3724	0.2686	4.6624	0.4080
	0.5		6.4016	0.3257	6.5344	0.3283
	1		11.047	0.3791	8.4369	0.2579
− 1	0.5	− 1	5.6810	0.4498	7.2588	0.1542
		− 0.5	5.8731	0.4288	4.3466	0.1785
		0	6.6866	0.3843	4.4028	0.2400
		0.5	7.8149	0.3256	7.5481	0.3285
		1	9.1038	0.2795	11.580	0.4031
0		− 1	3.7825	0.4501	6.4061	0.1540
		− 0.5	4.1789	0.4297	2.6391	0.1774
		0	5.3766	0.3860	2.6861	0.2377
		0.5	6.8262	0.3274	6.6696	0.3259
		1	8.3408	0.2806	10.995	0.4013
1		− 1	2.1064	0.4498	6.4898	0.1542
		− 0.5	3.0053	0.4289	2.6589	0.1783
		0	4.6818	0.3845	2.5253	0.2397
		0.5	6.4016	0.3257	6.5344	0.3283
		1	8.0726	0.2794	10.870	0.4031

**Table 3:** Computed values of SFC and LNN when $\delta = 0.8,\;\overline\omega = 0.5,\;Fr = 1,\;E = 0.2 = \varepsilon ,$ $\lambda_{1} = 1 = \lambda_{2} ,$ $Ra = 0.5,\;\theta_{r} = 0.5,\;Bi_{b} = 0.4,\;A_{a} = 0.5 = A_{b}$.

$\Omega$	$Re$	$Bi_{a}$	Lower disk		Upper disk	
			SFC	LNN	SFC	LNN
− 1	2	0.4	7.8149	0.3256	7.5481	0.3285
	5		8.5873	0.3308	7.8100	0.3355
	7		9.1059	0.3334	7.9928	0.3396
	10		9.8692	0.3360	8.2837	0.3456
0	2		6.8262	0.3274	6.6696	0.3259
	5		7.6137	0.3394	6.9564	0.3227
	7		8.1849	0.3478	7.1378	0.3181
	10		9.0527	0.3597	7.3950	0.3100
1	2		6.4016	0.3257	6.5344	0.3283
	5		7.2082	0.3319	7.4067	0.3337
	7		7.8075	0.3358	8.0267	0.3357
	10		8.7108	0.3412	8.9422	0.3376
− 1	2	0.1	8.2746	0.1252	7.9423	0.1259
		0.2	8.0541	0.2131	7.7514	0.2147
		0.3	7.9128	0.2772	7.6308	0.2795
		0.4	7.8149	0.3256	7.5481	0.3285
		0.5	7.7433	0.3634	7.4880	0.3666
0		0.1	7.2415	0.1254	6.9924	0.1243
		0.2	7.0431	0.2138	6.8359	0.2124
		0.3	6.9151	0.2785	6.7372	0.2770
		0.4	6.8262	0.3274	6.6696	0.3259
		0.5	6.7610	0.3656	6.6205	0.3640
1		0.1	6.7928	0.1252	6.8457	0.1258
		0.2	6.6057	0.2131	6.6945	0.2145
		0.3	6.4852	0.2772	6.5994	0.2793
		0.4	6.4016	0.3257	6.5344	0.3283
		0.5	6.3402	0.3635	6.4874	0.3664
0.5	2	0.1	6.7928	0.1252	6.8457	0.1258
	5		7.6470	0.1260	7.7191	0.1267
	7		8.2724	0.1265	8.3500	0.1270
	10		9.2090	0.1271	9.2891	0.1272
	2	0.3	6.4852	0.2772	6.5994	0.2793
	5		7.3037	0.2816	7.4712	0.2833
	7		7.9095	0.2843	8.0933	0.2848
	10		8.8208	0.2879	9.0138	0.2860
	2	0.5	6.3402	0.3635	6.4874	0.3664
	5		7.1375	0.3713	7.3603	0.3730
	7		7.7317	0.3764	7.9788	0.3757
	10		8.6286	0.3834	8.8906	0.3782

**Figure 2:**
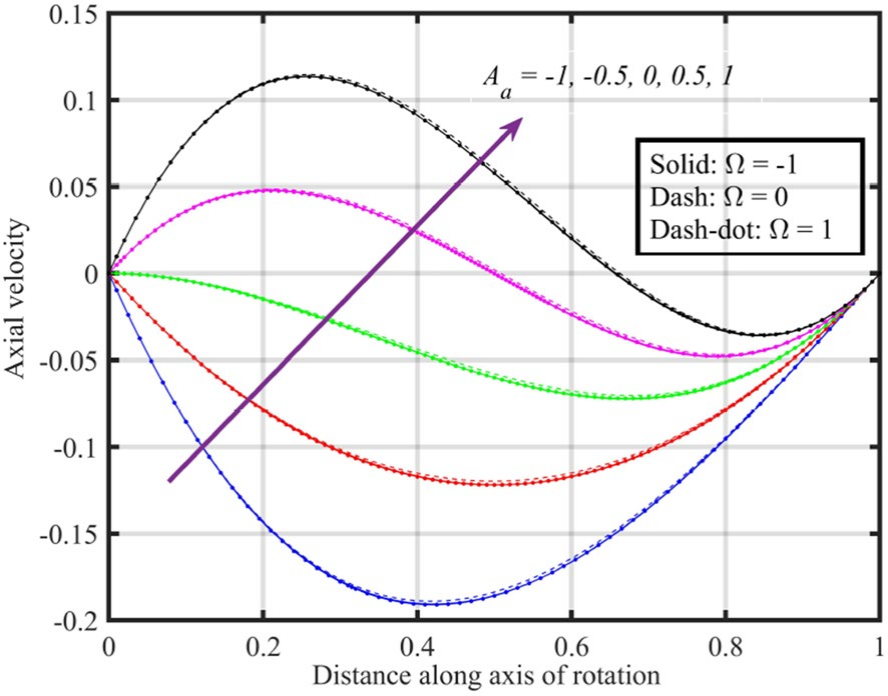
Consequences of varying $A_{a}$ on axial velocity $F\left( \xi \right)$ for distinct $\Omega$.

**Figure 3:**
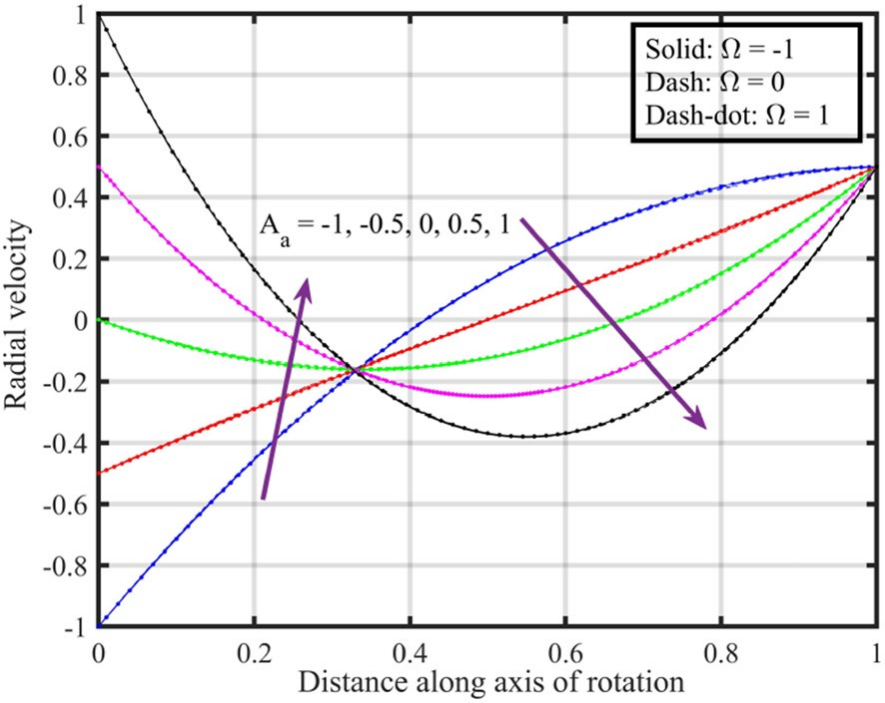
Consequences of varying $A_{a}$ on radial velocity $F^{\prime}\left( \xi \right)$ for distinct $\Omega$.

**Figure 4:**
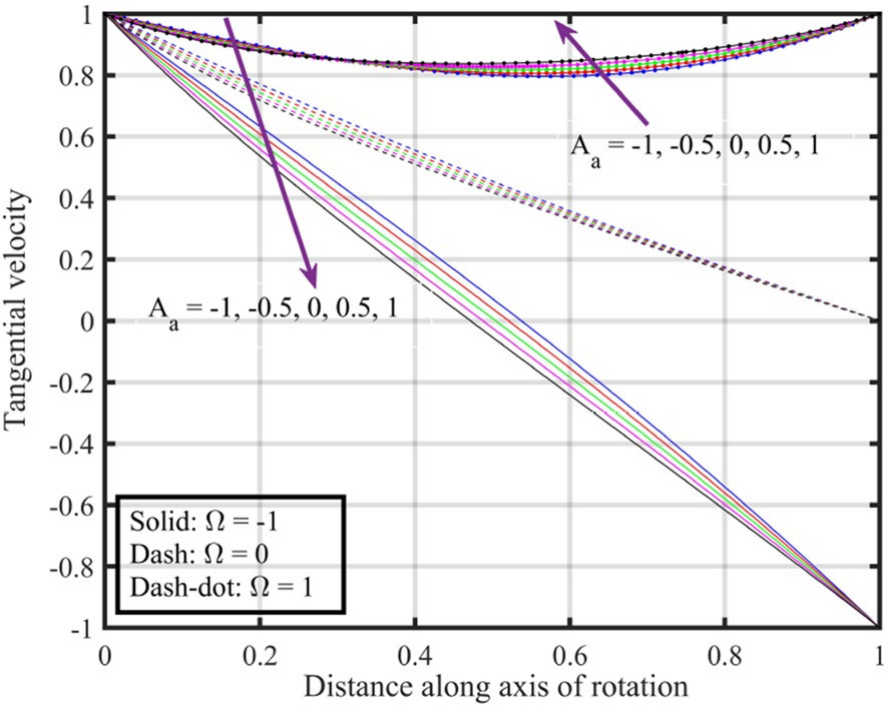
Consequences of varying $A_{a}$ on tangential velocity $G\left( \xi \right)$ for distinct $\Omega$.

**Figure 5:**
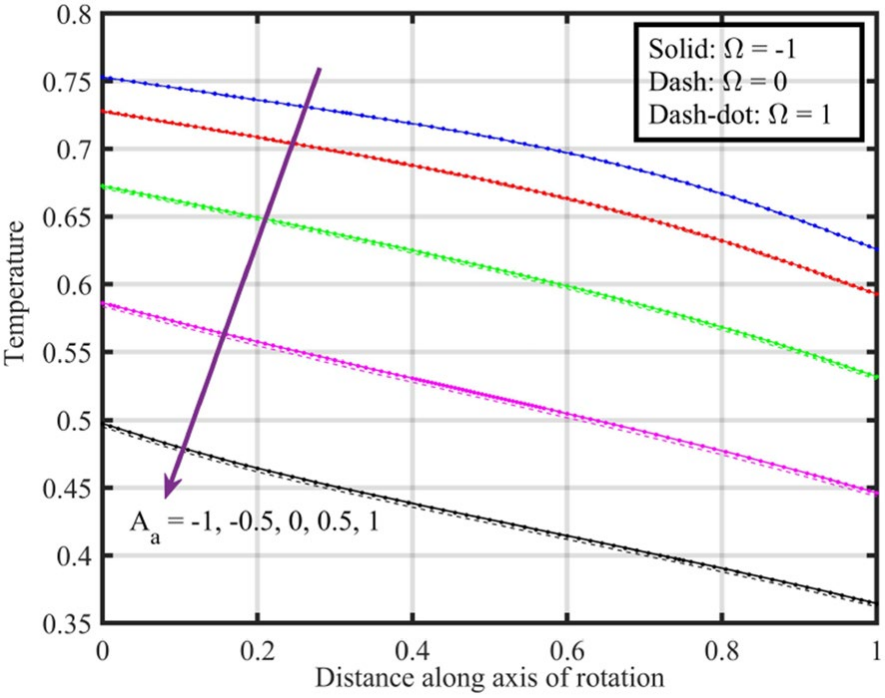
Consequences of varying $A_{a}$ on temperature $\theta \left( \xi \right)$ for distinct $\Omega$.

**Figure 6:**
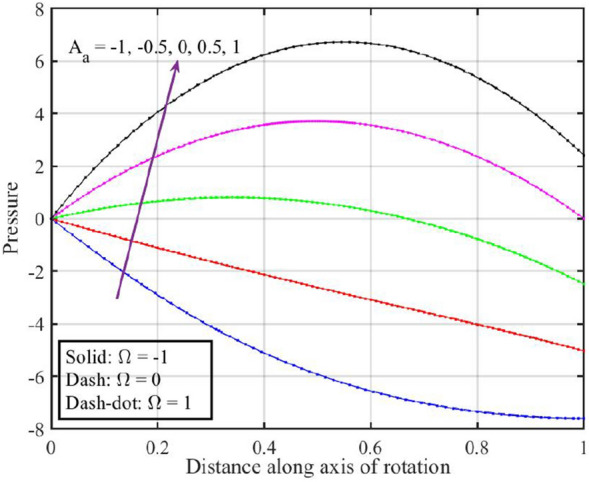
Consequences of varying $A_{a}$ on pressure $P\left( \xi \right)$ for distinct $\Omega$.

Figure 2 shows that increasing the lower disk stretching parameter $A_{a}$, leads to an increase in the axial velocity $F\left( \xi \right)$ for each $\Omega$. This could be attributed to the fact that as the stretching parameter increases, the lower disk's deformation causes changes in the flow pattern. This alteration may result in an increased $F\left( \xi \right)$, possibly due to changes in the effective area through which the fluid flows. Moreover, it is noticed that axial velocity is higher for the case $\Omega = 0$ followed by $\Omega = - 1$ and $1$. Figure 3 that the rise in $A_{a}$ results enhancement of the radial velocity $F^{\prime}\left( \xi \right)$ at the lower disk, but near the upper disk the velocity gets declined. This can be explained by the stretching effect causing changes in the radial distribution of velocities. The enhancement at the lower disk could be due to increased surface effects influencing the radial flow, while near the upper disk, other factors might be dominating and causing a decline. Figure 4 implies that, for $\Omega = - 1$ and $0$ tangential velocity $G\left( \xi \right)$ decrease with growing $A_{a}$ parameter while a dual behavior is recorded for $\Omega = 1$, here for $\xi < 0.3$ velocity decreases while it increases for $\xi > 0.3$. Here, negative values of velocity indicate that movement of upper disk is faster than the lower disk. Figures 5 and 6 specify that a reduction is temperature profile while an escalation in pressure distribution is recorded for growing values of $A_{a}$. This is because of the changes in flow patterns and energy dissipation associated with the increased stretching effect.

**Figure 7:**
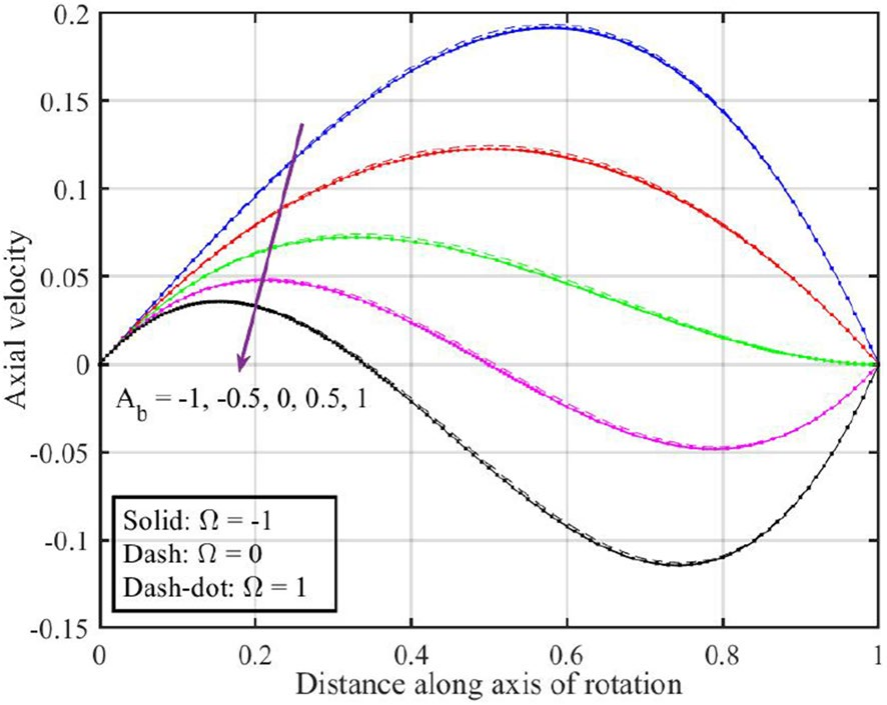
Consequences of varying $A_{b}$ on axial velocity $F\left( \xi \right)$ for distinct $\Omega$.

**Figure 8:**
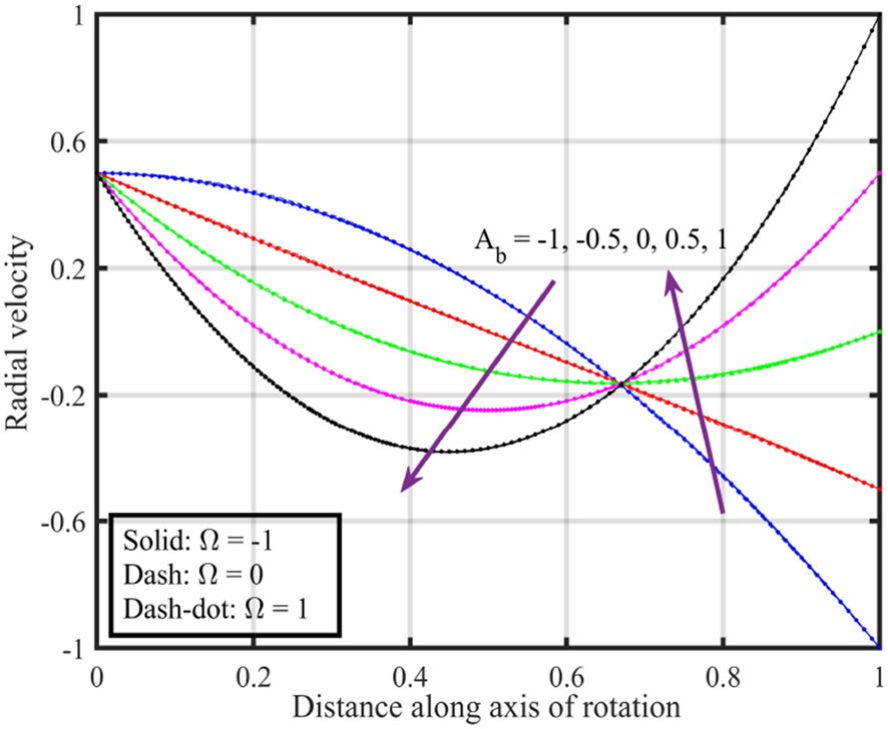
Consequences of varying $A_{b}$ on radial velocity $F^{\prime}\left( \xi \right)$ for distinct $\Omega$.

**Figure 9:**
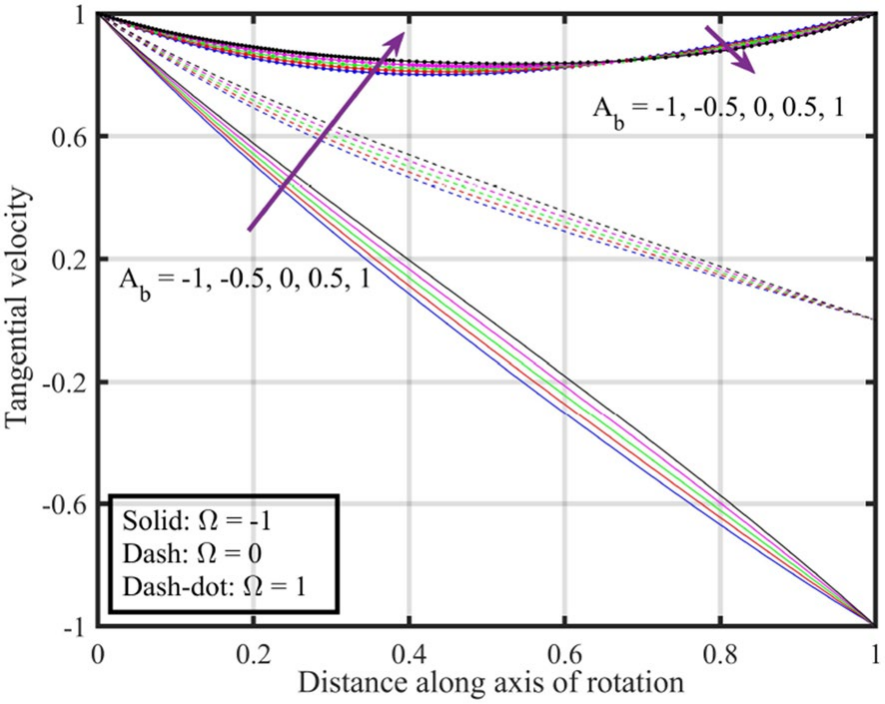
Consequences of varying $A_{b}$ on tangential velocity $G\left( \xi \right)$ for distinct $\Omega$.

**Figure 10:**
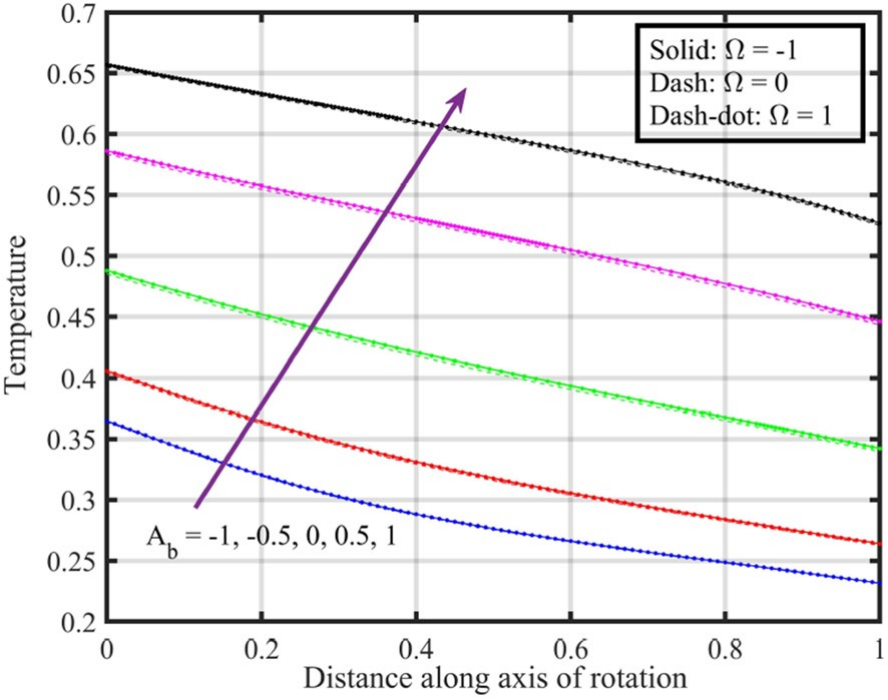
Consequences of varying $A_{b}$ on temperature $\theta \left( \xi \right)$ for distinct $\Omega$.

**Figure 11:**
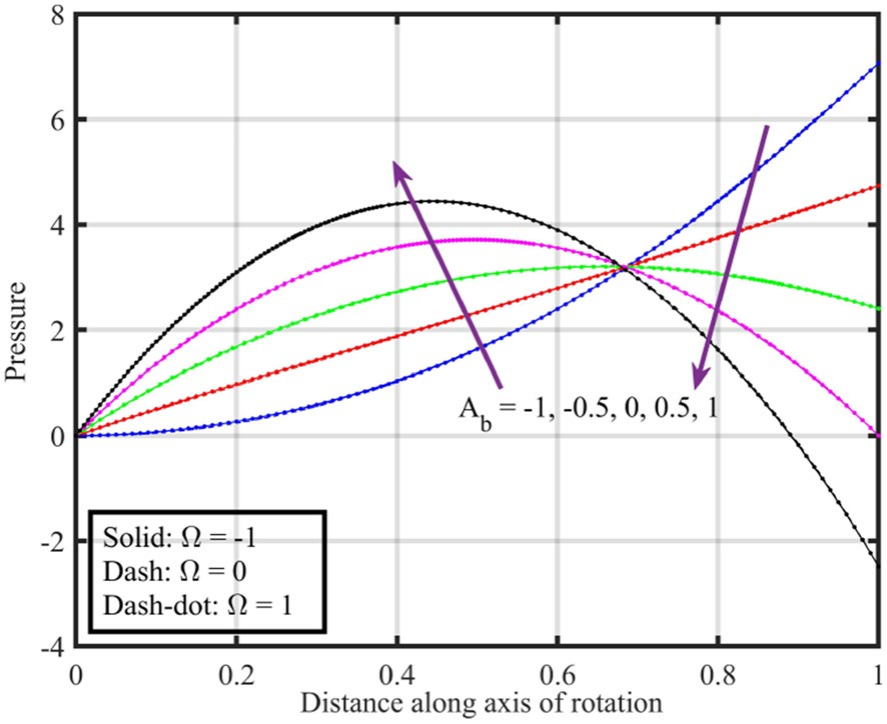
Consequences of varying $A_{b}$ on pressure $P\left( \xi \right)$ for distinct $\Omega$.

Figures 7, 8, 9, 10, 11 are plotted to highlight the impacts of altering $A_{b}$ on flow properties (velocities, temperature and pressure distribution) for co-rotation $\left( {\;\Omega = 1} \right)$, rotor–stator $\left( {\;\Omega = 0} \right)$, and counter rotation $\left( {\;\Omega = - 1} \right)$ situations, respectively. Figures show that an increase in the values of $A_{b}$ from $- 1$ to $1$, results in a decrease in the axial velocity (see Fig. 7). Physically, increasing $A_{b}$ causes changes in the geometry of upper disk and surface effects, also the disks are assumed to be rotating about their axis, thus results in a decrease in $F\left( \xi \right)$ . Radial velocity declines when $\xi < 0.6$ while the opposite pattern is recorded for $\xi > 0.6$ (see Fig. 8), and tangential velocity $G\left( \xi \right)$ increases for $\Omega = - 1$ and $0$ cases, while dual behavior is observed for $\Omega = 1$, for $\xi > 0.8$ which velocity decreases whereas it increases for $\xi < 0.8$ (see Fig. 9). Figure 10 denotes that the temperature profile escalates with growth in; here, for a particular fluid temperature, it doesn’t show any significant variation for $\Omega = 1,\,0,\; - 1$. Physically, increasing $A_{b}$ leads to enhanced mixing and dissipation of energy, resulting in an overall increase in the temperature profile. and Fig. 11 shows that for $\xi < 0.7$ the pressure is in a positive direction and increases with an increase in the values of $A_{b}$, whereas it falls in the remaining region. The positive pressure and its increase in a specific region might be attributed to changes in the flow patterns and dynamic forces caused by alterations in $A_{b}$. The falling pressure in other regions may result from the counteracting effects of the stretching parameter on the flow dynamics.

**Figure 12:**
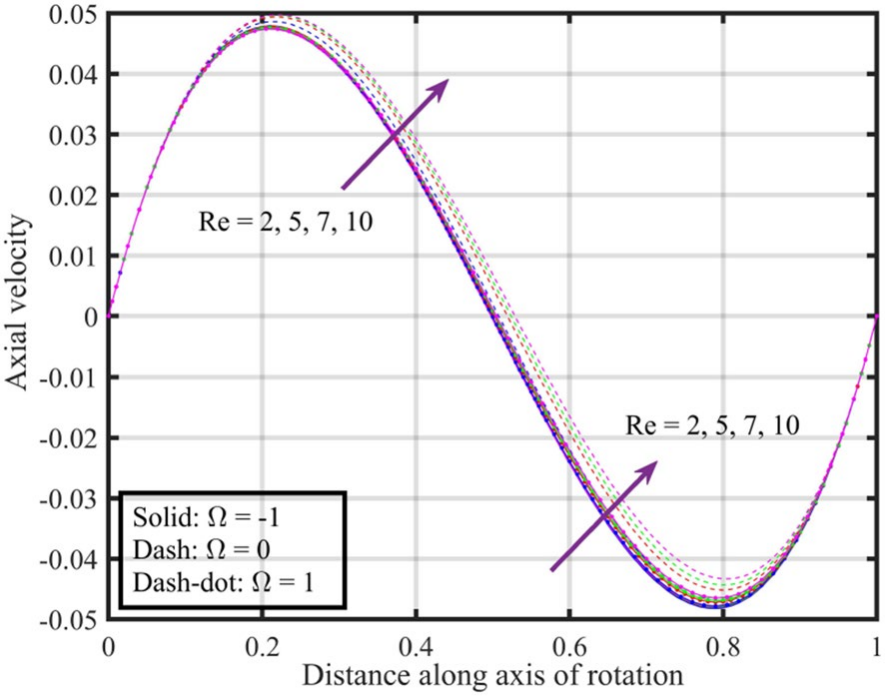
Consequences of varying $Re$ on axial velocity $F\left( \xi \right)$ for distinct $\Omega$.

**Figure 13:**
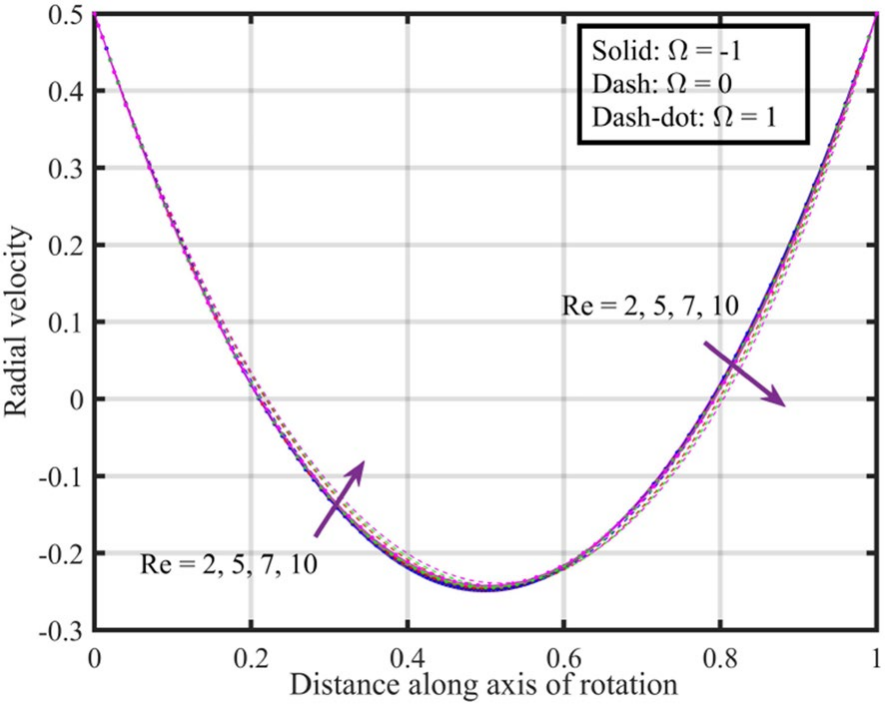
Consequences of varying $Re$ on radial velocity $F^{\prime}\left( \xi \right)$ for distinct $\Omega$.

**Figure 14:**
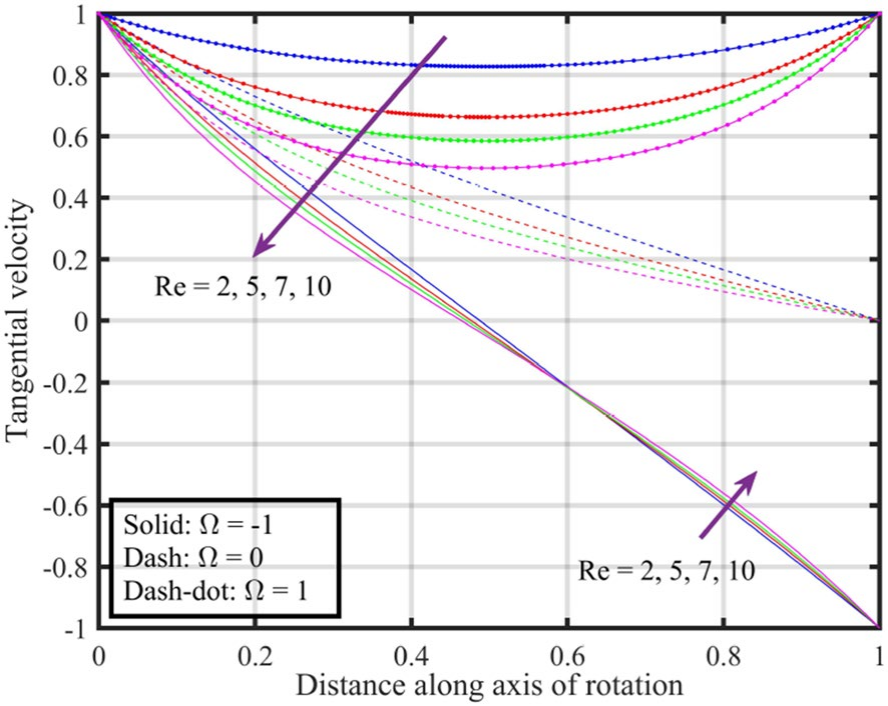
Consequences of varying $Re$ on tangential velocity $G\left( \xi \right)$ for distinct $\Omega$.

**Figure 15:**
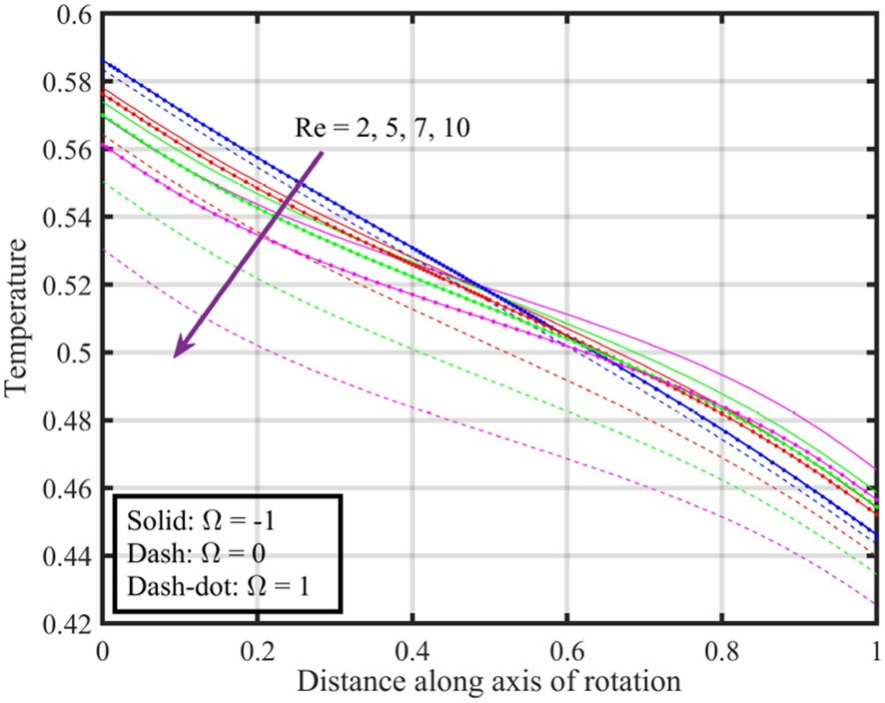
Consequences of varying $Re$ on temperature $\theta \left( \xi \right)$ for distinct $\Omega$.

**Figure 16:**
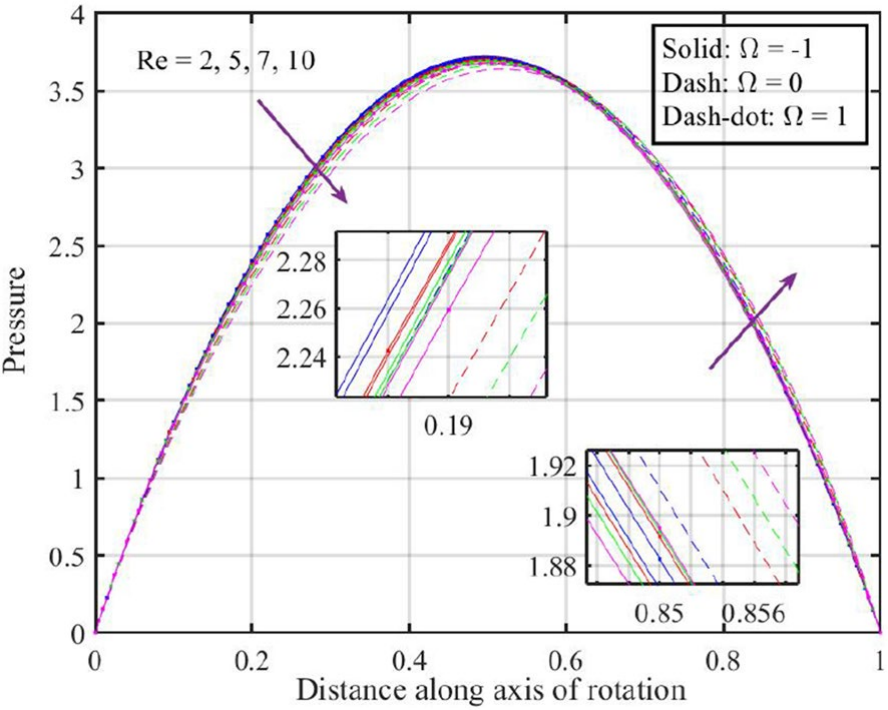
Consequences of varying $Re$ on pressure $P\left( \xi \right)$ for distinct $\Omega$.

Figures 12, 13, 14, 15, 16 are shown in order to visually depict the implications of different $Re$ on flow characteristics, such as velocities, temperature, and pressure distribution, under the scenarios of co-rotation $\left( {\;\Omega = 1} \right)$, rotor–stator $\left( {\;\Omega = 0} \right)$, and counter-rotation $\left( {\;\Omega = - 1} \right)$. Axial velocity $F\left( \xi \right)$ of the fluid increases with increase in values of $Re$ for each value of $\Omega$ but velocity correspond to $\left( {\;\Omega = 1} \right)$ is superior than other cases, further near the lower and upper disks the variation in fluid velocity is negligible, as depicted in Fig. 12. Physically, higher $Re$ typically indicate an increase in fluid inertia compared to viscous forces. This leads to more streamlined and faster flow, resulting in an increase in $F\left( \xi \right)$. Moreover, the negligible variation in $F\left( \xi \right)$ near lower and upper disks may be due to the influence of boundary conditions, geometric constraints, or other factors that limit the impact of Reynolds number on the flow near these surfaces. Figure 13 illustrates that $F^{\prime}\left( \xi \right)$ of the fluid is a monotonically increasing function of $Re$ for $\xi < 0.6$, and behave as decreasing function in the remaining region. Variation in tangential velocity due to distinct $Re$ is shown in Fig. 14, figure shows that for $\Omega = 0$ and $1$, velocity reduces but for $\Omega = - 1$ it reduces for $\xi < 0.6$ and thereafter it increases. Figure 15 shows that fluid is hotter at the surface of lower disk compared to upper disk, fluid temperature reduces with growth in Reynolds number from $2$ to $10$ for each case of $\Omega$ while for $\xi > 0.6$ a contrasting trend is observed is noticed for temperature profile correspond to $\;\Omega = - 1$ and $1$. And, pressure reduces with growing $Re$ for $\xi < 0.6$ and in the remaining region it elevates. The reduction in pressure might be associated with enhanced kinetic energy and turbulence at higher Reynolds numbers, while the increase in pressure in other cases could be influenced by the specific rotational configurations and their effects on flow patterns and dynamic forces.

**Figure 17:**
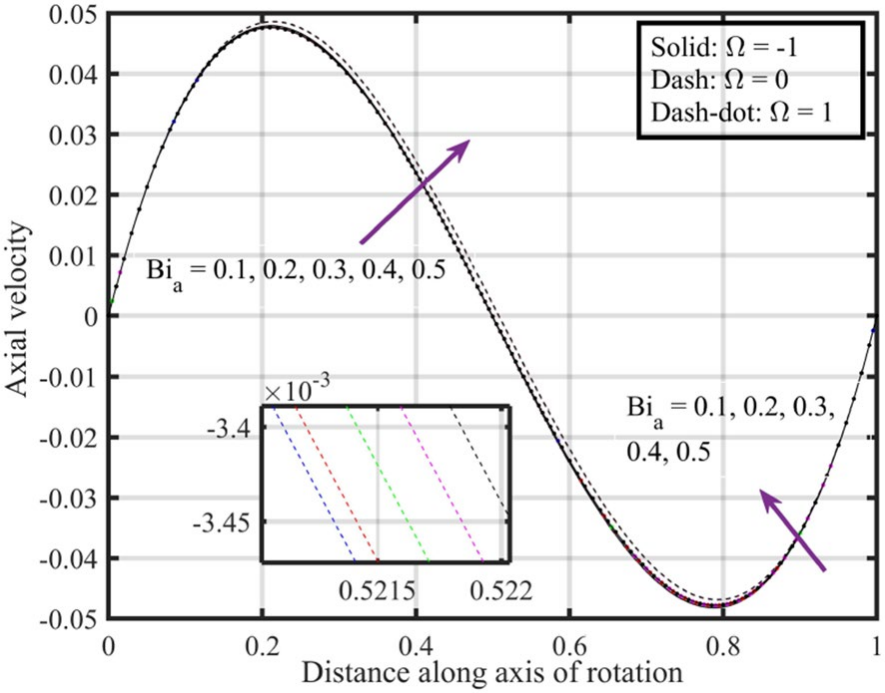
Consequences of varying $Bi_{a}$ on axial velocity $F\left( \xi \right)$ for distinct $\Omega$.

**Figure 18:**
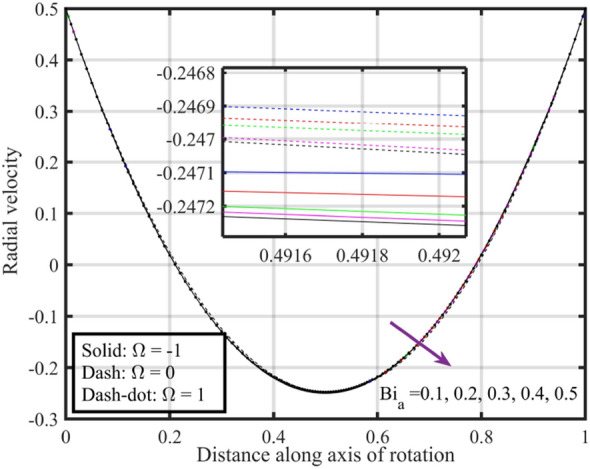
Consequences of varying $Bi_{a}$ on radial velocity $F^{\prime}\left( \xi \right)$ for distinct $\Omega$.

**Figure 19:**
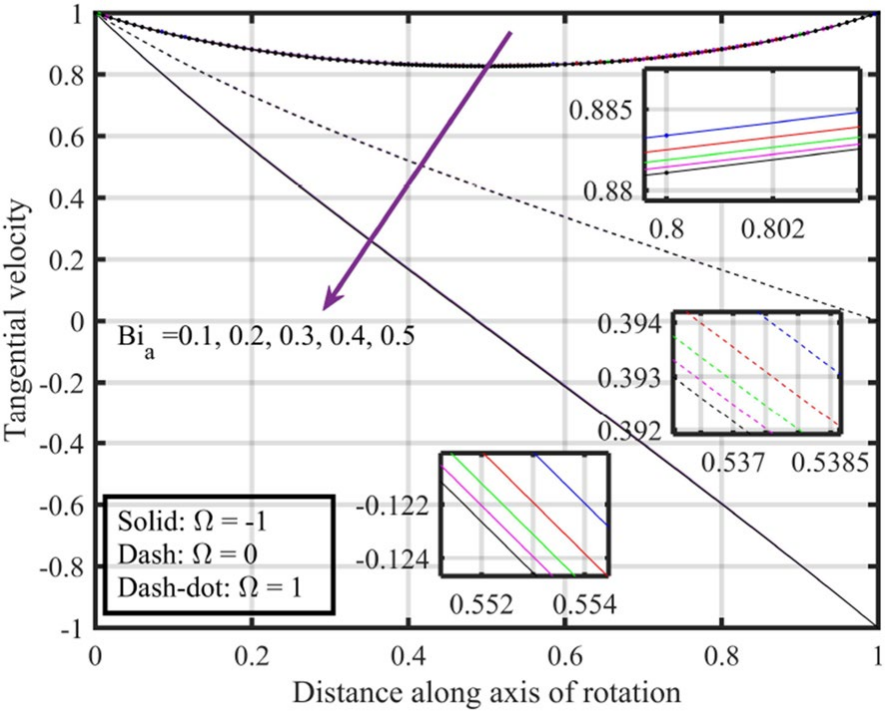
Consequences of varying $Bi_{a}$ on tangential velocity $G\left( \xi \right)$ for distinct $\Omega$.

**Figure 20:**
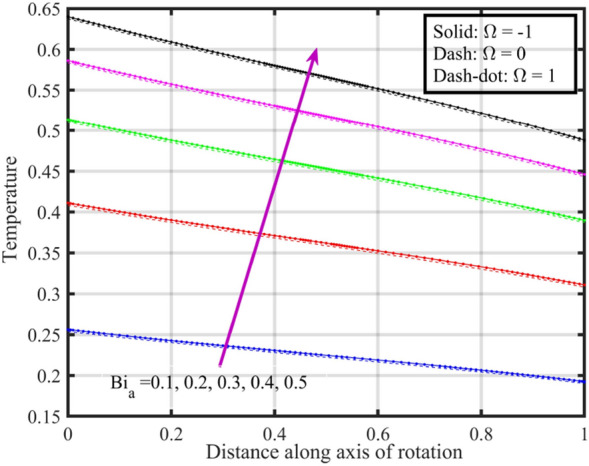
Consequences of varying $Bi_{a}$ on temperature $\theta \left( \xi \right)$ for distinct $\Omega$.

**Figure 21:**
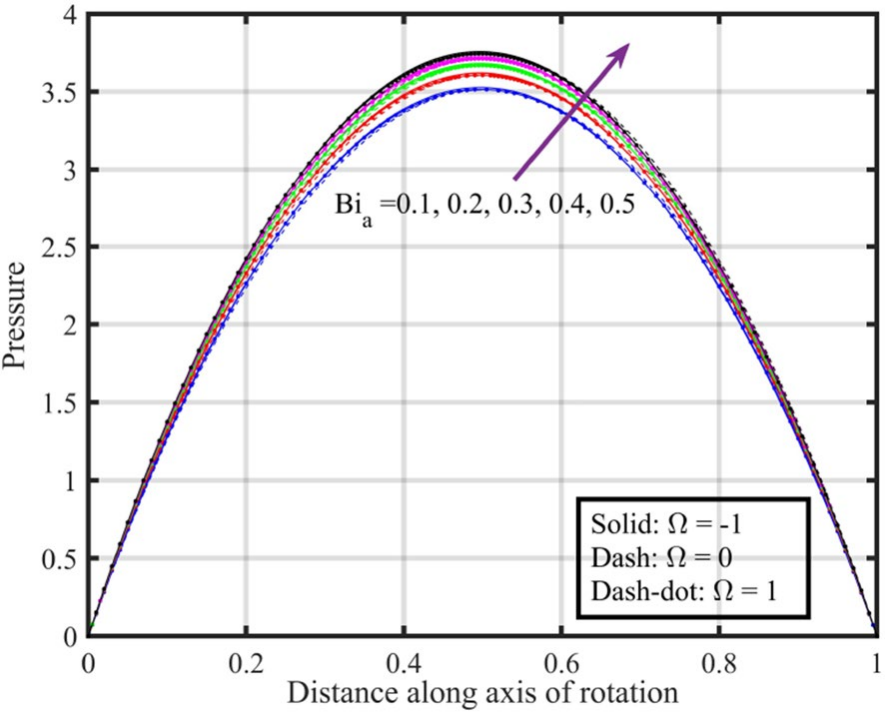
Consequences of varying $Bi_{a}$ on pressure $P\left( \xi \right)$ for distinct $\Omega$.

Figures 17, 18, 19, 20, 21 show the effects of changing $Bi_{a}$ on flow properties (velocities, temperature, and pressure distribution) for co-rotation $\left( {\;\Omega = 1} \right)$, rotor–stator $\left( {\;\Omega = 0} \right)$, and counter-rotation scenarios. Figure 17 illustrates that axial velocity near the surface of the upper and lower discs has insignificant variation with distinct values of $Bi_{a}$ and $\Omega$, but for $\xi > 0.2$ velocity field increases with increasing $Bi_{a}$, and velocity becomes negative for $\xi \ge 0.5$. The stability of axial velocity for lower values of $\xi$ suggests that at these regions, the heat transfer effects do not significantly influence the axial flow. And, increase in velocity for $\xi > 0.2$ may be indicative of stronger interactions between heat transfer and fluid dynamics, leading to changes in axial flow patterns. The negative velocity for $\xi \ge 0.5$ indicate a reverse flow or backflow in certain regions. Radial velocity $F^{\prime}\left( \xi \right)$ and tangential velocity $G\left( \xi \right)$ reduce with growth in $Bi_{a}$, see Figs. 18 and 19, respectively. The reduction in $F^{\prime}\left( \xi \right)$ and $G\left( \xi \right)$ with increasing $Bi_{a}$ is likely a consequence of enhanced heat transfer effects, higher $Bi_{a}$ indicate a stronger coupling between the fluid and the solid surfaces, impacting the radial and tangential components of the flow. Figure 20 illustrate that near the lower disc, fluid is hotter compared to fluid at upper disc and fluid temperature upsurges with growth in $Bi_{a}$; similar behavior is recorded for pressure profiles see Fig. 21. Higher $Bi_{a}$ likely lead to variations in pressure due to the intensified heat transfer effects on the fluid.

**Figure 22:**
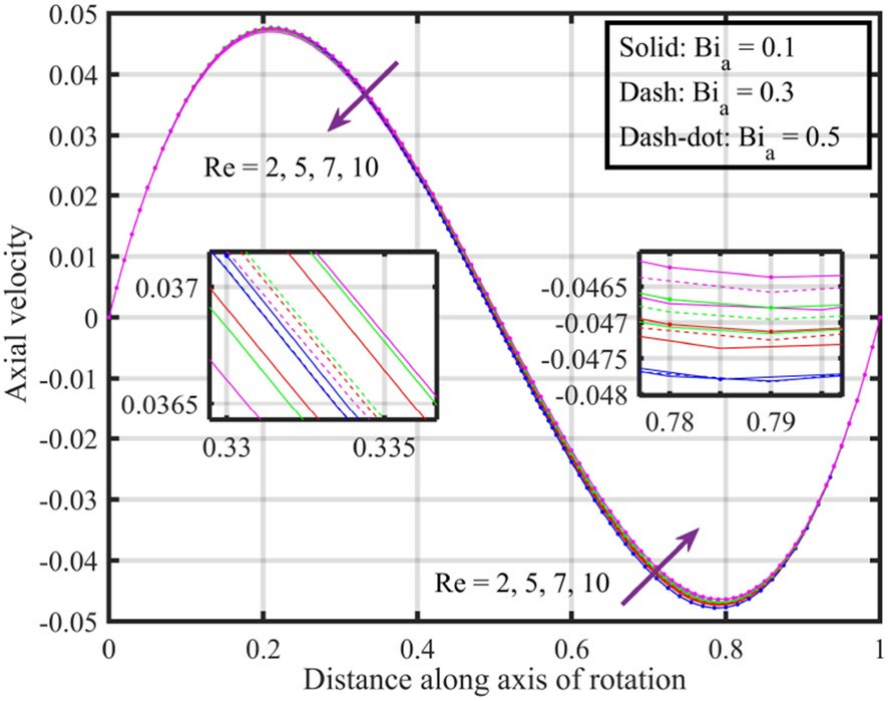
Consequences of varying $Re$ on axial velocity $F\left( \xi \right)$ for distinct $Bi_{a}$.

**Figure 23:**
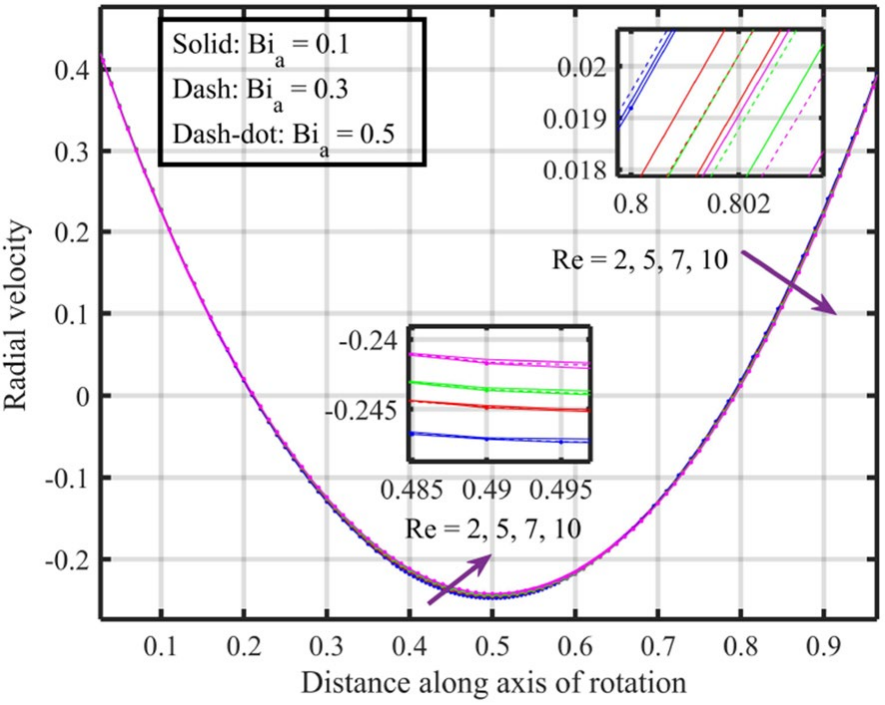
Consequences of varying $Re$ on radial velocity $F^{\prime}\left( \xi \right)$ for distinct $Bi_{a}$.

**Figure 24:**
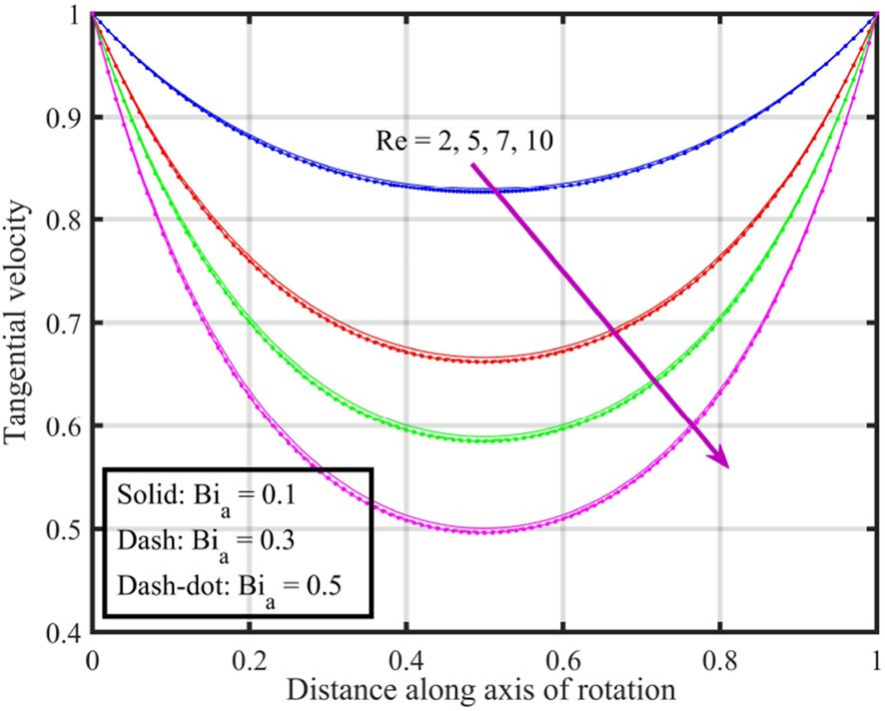
Consequences of varying $Re$ on tangential velocity $G\left( \xi \right)$ for distinct $Bi_{a}$.

**Figure 25:**
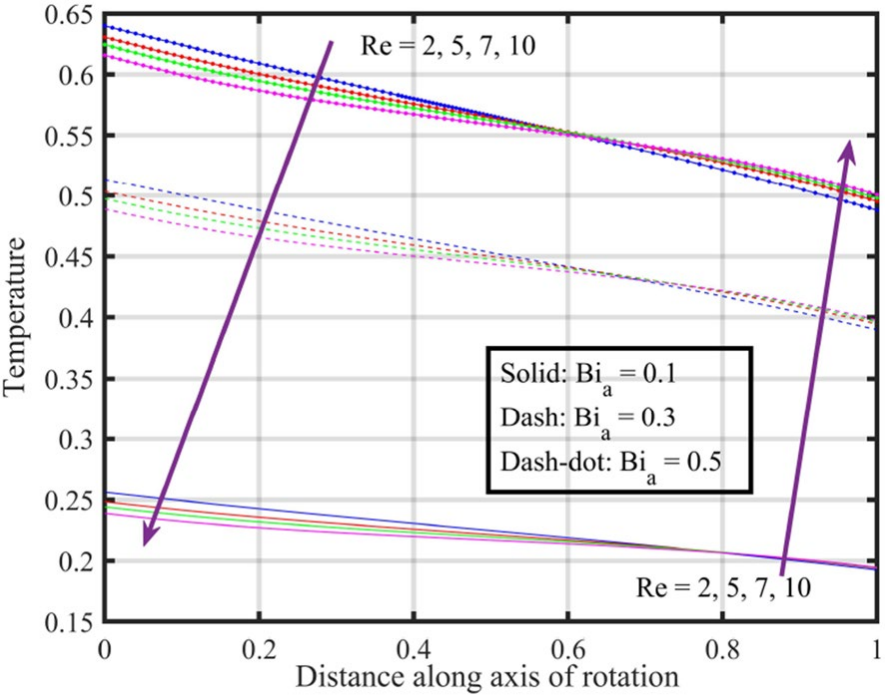
Consequences of varying $Re$ on temperature $\theta \left( \xi \right)$ for distinct $Bi_{a}$.

**Figure 26:**
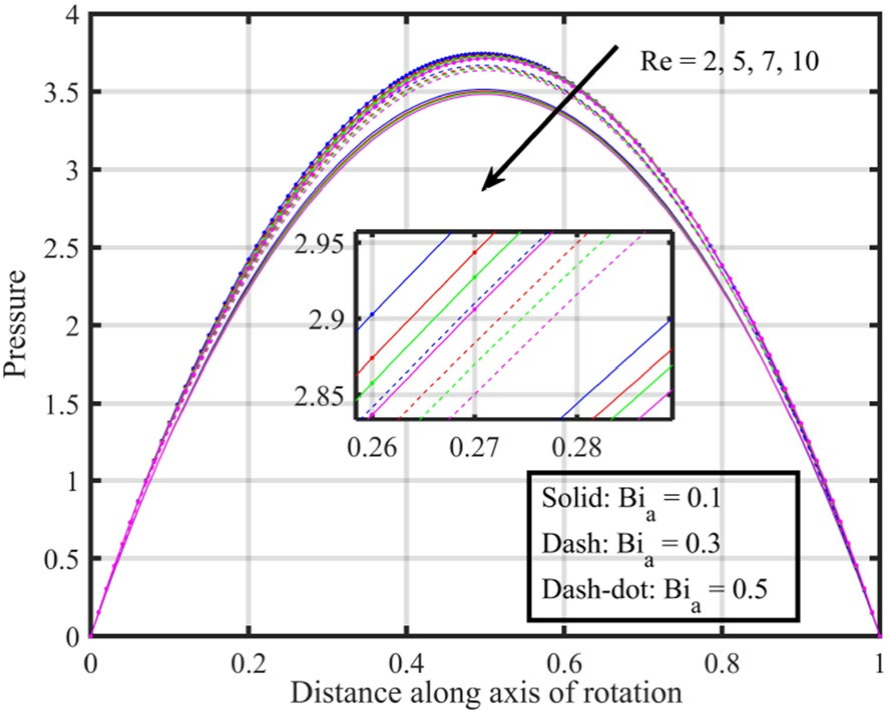
Consequences of varying $Re$ on pressure $P\left( \xi \right)$ for distinct $Bi_{a}$.

Figures 22, 23, 24, 25, 26 plotted to illustrate the consequences of varying $Re$ on flow features (velocities, temperature and pressure distribution) for distinct values of $Bi_{a}$. Figure 22 shows that near the lower and upper discs the difference between axial velocities is insignificant for distinct $Re$, but for $0.2 < \xi < 0.4$ it decreases with growth in $Re$ while for $0.4 < \xi < 0.8$ it rises. Figure 23 illustrate that, for a particular $Re$ radial velocity decreases for $\xi < 0.5$ while it rises thereafter, however the distribution of radial velocity does not show any significant variation with distinct values of $Re$ for $\xi < 0.3$ whereas for $0.3 < \xi < 0.6$ it increases and for $\xi > 0.75$ it reduces. Figure 24 shows that, at both lower and upper disk, tangential velocity is higher, and velocity distribution decreases with increase in Reynolds number. Usually, higher tangential velocity near the discs is likely due to the rotational motion of the system, which is more pronounced near the rotating surfaces; and the decrease in velocity distribution with an increase in Reynolds number is because of the changes in the flow dynamics associated with different Reynolds numbers. Figure 25 shows that, near the lower disk, fluid have higher temperature, and as temperature falls as fluid moves away from lower disk, as it reaches the upper disk fluid temperature elevates, thus higher temperature near the lower disc indicates more efficient heat transfer from the solid surfaces to the fluid in that region. Moreover, fluid temperature corresponds to $Bi_{a} = 0.5$ is higher compared to $Bi_{a} = 0.3$ and $0.1$; this suggest that the heat transfer characteristics are influenced by the thermal properties of the system. Pressure distribution profiles elucidate that it is minimum at the extreme ends i.e., lower and upper discs while it is maximum at the mid-way of the discs; moreover, it reduces with escalation in $Re$ and distribution profile correspond to $Bi_{a} = 0.5$ is higher followed by $Bi_{a} = 0.3$ and $0.1$, respectively, see Fig. 26.

It is noted from Table 2 that as the lower disc undergoes the transition from radial shrinking to stretching, the lower disc has a higher SFC, whereas the LNN is higher at the upper disc; the opposite pattern is recorded when the upper disc undergoes a similar transition while the lower disc is radially stretching. It is anticipated from Table 3 that, when discs are in co-rotation, the LNN at the lower disc is higher compared to the upper disc for a higher Reynolds number, whereas the upper disc has a higher SFC than the lower disc; on the other hand, for counter-rotation and no rotation, the lower disc has a higher SFC. For the case where the lower disc is exposed to convective heating, it is noted from Table 3 that for each case of $\Omega$, the upper disc has a higher local Nusselt number; however, the variation between LNN for these discs is less than 1%. A similar pattern is recorded for LNN when discs are in co-rotation and $Re$ and $Bi_{a}$ are varying; here, both LNN and SFC increase with increasing magnitudes of $Re$ and $Bi_{a}$.

In the case of counter rotation of discs, Table 4 presents the variations of LNN (local Nusselt number) and SFC (skin friction coefficient) concerning the Casson factor, thermal conductivity parameter, and viscosity variation parameter. From the results, it is evident that increasing the Casson parameter from 0.25 to 1.5 leads to a significant reduction in SNF (skin friction) on both lower and upper discs, by up to 65%. However, the increase in LNN is minimal, being less than 0.06%. Similarly, augmenting the viscosity variation parameter from 0 to 0.2 results in a decrease in SFC of up to 13%. Conversely, an increase in the thermal conductivity parameter exhibits a notable impact on LNN, affecting both lower and upper discs, with a change of up to 17% in LNN with a variation in the thermal conductivity parameter from 0 to 0.6.

**Table 4:** Computed values of SFC and LNN when $\Omega =Fr={\lambda }_{1}={\lambda }_{2}=1$, $Re=Ra=2, Pr=6.2$, ${\theta }_{r}=0.5$, ${A}_{a}={A}_{b}=0.5, {Bi}_{a}={Bi}_{b}=0.4$

$\delta$	$\varepsilon$	$E$	Lower disk		Upper disk	
			SFC	LNN	SFC	LNN
0.25	0.2	0.2	14.0307	0.7881	14.1633	0.7932
0.75			6.6229	0.7884	6.7361	0.7927
0.80			6.3918	0.7884	6.5044	0.7927
1.00			5.6990	0.7885	5.8093	0.7926
1.50			4.7763	0.7886	4.8834	0.7924
0.80	0.2	0	7.0701	0.7891	7.1631	0.7914
		0.1	6.7136	0.7887	6.8174	0.7921
		0.2	6.3918	0.7884	6.5044	0.7927
		0.3	6.1000	0.7881	6.2195	0.7933
0.8	0	0.2	6.3775	0.8452	6.4992	0.8502
	0.2		6.3918	0.7884	6.5044	0.7927
	0.4		6.4030	0.7419	6.5078	0.7457
	0.6		6.4121	0.7031	6.5102	0.7063

## Summary and conclusion

In this investigation, we have conducted a comprehensive examination of how various parameters influence fluid dynamics and heat transfer between two parallel disks. Our study provides a visual representation of how these parameters impact fluid velocity, encompassing axial, radial, and tangential components, as well as temperature distribution and pressure profiles along the axis of rotation. The quantitative dimensions of our research is encapsulated in the analysis of key metrics, namely the Skin Friction Coefficient (SFC) and the Local Nusselt Number (LNN), for both the lower and upper disks. We have also explored the behavior of SFC and LNN as the lower and upper disks transition between radial shrinking and stretching.

From these investigations, several salient conclusions emerge:

- The Casson parameter plays a crucial role in reducing the skin friction coefficients on the rotating disks to a very high extent, while the thermal conductivity parameter primarily impacts the heat transfer rate (LNN) with minimal effect on the skin friction coefficient.
- During the transition from radial shrinking to stretching of the lower disc, we observed a higher Skin Friction Coefficient (SFC) for the lower disc, while the Local Nusselt Number (LNN) is elevated at the upper disc. Conversely, when the upper disc undergoes a similar transition while the lower disc is radially stretching, the roles are reversed, with the lower disc exhibiting a higher SFC and the upper disc displaying a higher LNN.
- In scenarios involving co-rotation, the LNN at the lower disc exceeds that at the upper disc for higher Reynolds numbers. However, it is consistently noted that the upper disc maintains a higher SFC than the lower disc under identical conditions.
- In cases of counter-rotation and no rotation, the lower disc consistently demonstrates a higher SFC compared to the upper disc.
- When convective heating is applied to the lower disc, the upper disc registers a higher Local Nusselt Number. Nevertheless, the variation in LNN between these two discs remains minimal, at less than 1%.

These findings collectively contribute to a deeper understanding of the dynamic behavior of parallel rotating disks under diverse circumstances. They are of significant relevance to engineers and researchers working on systems involving rotating components, providing essential insights into optimizing performance and heat transfer management.

Finally, fluid flow between coaxial disks may have industrial uses, such as the construction of mixing devices, lubricating systems, and cooling systems. Future study might concentrate on improving these applications' efficiency, sustainability, and cost-effectiveness.

## Data Availability

All data generated or analysed during this study are included in this published article.

## References

[1] Kármán TV (1921). Über laminare und turbulente Reibung. ZAMM-J. Appl. Math. Mech./Zeitschrift für Angewandte Mathematik und Mechanik.

[2] Batchelor GK (1951). Note on a class of solutions of the Navier-Stokes equations representing steady rotationally-symmetric flow. Q. J. Mech. Appl. Math..

[3] Stewartson, K. On the flow between two rotating coaxial disks. In *Mathematical Proceedings of the Cambridge Philosophical Society*, vol. 49, no. 2, 333–341 (Cambridge University Press, 1953).

[4] Holodniok M, Kubicek M, Hlavacek V (1977). Computation of the flow between two rotating coaxial disks. J. Fluid Mech..

[5] Crewther I, Huilgol RR, Jozsa R (1991). Axisymmetric and non-axisymmetric flows of a non-Newtonian fluid between coaxial rotating discs. Philos. Trans. R. Soc. Lond. Ser. A Phys. Eng. Sci..

[6] Rajagopal KR (1992). Flow of viscoelastic fluids between rotating disks. Theor. Comput. Fluid Dyn..

[7] Asghar S, Hanif K, Hayat T, Khalique CM (2007). MHD non-Newtonian flow due to non-coaxial rotations of an accelerated disk and a fluid at infinity. Commun. Nonlinear Sci. Numer. Simul..

[8] Ghosh SK, Bég OA, Narahari M (2009). Hall effects on MHD flow in a rotating system with heat transfer characteristics. Meccanica.

[9] Aus der Wiesche S, Kulacki F (2017). Heat transfer in rotating flows. Handbook of Thermal Science and Engineering.

[10] Ahmed J, Khan M, Ahmad L (2019). MHD swirling flow and heat transfer in Maxwell fluid driven by two coaxially rotating disks with variable thermal conductivity. Chin. J. Phys..

[11] Upadhya SM, Devi RR, Raju CSK, Ali HM (2021). Magnetohydrodynamic nonlinear thermal convection nanofluid flow over a radiated porous rotating disk with internal heating. J. Therm. Anal. Calorim..

[12] Nayak MK, Shaw S, Khan MI, Makinde OD, Chu YM, Khan SU (2021). Interfacial layer and shape effects of modified Hamilton’s Crosser model in entropy optimized Darcy–Forchheimer flow. Alex. Eng. J..

[13] Wang Y, Zhang L, Liu H, Yin L, Xiao Y, Liu Y, Zeng Z (2022). Instabilities of thermocapillary flows in large Prandtl number liquid bridges between two coaxial disks with different radii. Phys. Fluids.

[14] Wang Y, Zeng Z, Liu H, Zhang L, Yin L, Xiao Y, Liu Y (2022). Flow instabilities in thermocapillary liquid bridges between two coaxial disks with different radii. Int. J. Heat Mass Transf..

[15] Vijay N, Sharma K (2022). Heat and mass transfer study of ferrofluid flow between co-rotating stretchable disks with geothermal viscosity: Ham analysis. Chin. J. Phys..

[16] Hussain T, Xu H (2022). Time-dependent squeezing bio-thermal MHD convection flow of a micropolar nanofluid between two parallel disks with multiple slip effects. Case Stud. Therm. Eng..

[17] Mehdi I, Abbas Z, Hasnain J (2022). MHD flow and heat transfer between two rotating disks under the effects of nanomaterials (MoS2) and thermal radiation. Case Stud. Therm. Eng..

[18] Chhabra RP (2010). Non-Newtonian fluids: an introduction. Rheology of Complex Fluids.

[19] Böhme G (2012). Non-Newtonian Fluid Mechanics.

[20] Upreti H, Pandey AK, Uddin Z, Kumar M (2022). Thermophoresis and Brownian motion effects on 3D flow of Casson nanofluid consisting microorganisms over a Riga plate using PSO: A numerical study. Chin. J. Phys..

[21] Raza A, Khan SU, Farid S, Khan MI, Sun TC, Abbasi A, Khan MI, Malik MY (2021). Thermal activity of conventional Casson nanoparticles with ramped temperature due to an infinite vertical plate via fractional derivative approach. Case Stud. Therm. Eng..

[22] Raza A, Khan SU, Al-Khaled K, Khan MI, Haq AU, Alotaibi F, Abd Allah AM, Qayyum S (2022). A fractional model for the kerosene oil and water-based Casson nanofluid with inclined magnetic force. Chem. Phys. Lett..

[23] Raza, A., Khan, S. U., Thumma, T. & Haq, A. U. Fractional simulations for slip flow of Casson CMC-CNTs hybrid nanofluid with Mittag-Leffler kernel and Prabhakar fractional simulations. *Waves Random Complex Media* **33**, 1–17. 10.1080/17455030.2023.2226747 (2023).

[24] Lin Y, Raza A, Khan U, Nigar N, Elattar S, AlDerea AM, Khalifa HAEW (2023). Prabhakar fractional simulation for thermal and solutal transport analysis of a Casson hybrid nanofluid flow over a channel with buoyancy effects. J. Magn. Magn. Mater..

[25] Mohyud-Din ST, Khan SI (2016). Nonlinear radiation effects on squeezing flow of a Casson fluid between parallel disks. Aerospace Sci. Technol..

[26] Rafiq S, Nawaz M, Mustahsan M (2018). Casson fluid flow due to non-coaxial rotation of a porous disk and the fluid at infinity through a porous medium. J. Appl. Mech. Tech. Phys..

[27] Hayat T, Khan MWA, Khan MI, Waqas M, Alsaedi A (2018). Impact of chemical reaction in fully developed radiated mixed convective flow between two rotating disk. Phys. B Condens. Matter.

[28] Ramesh K, Ojjela O, Nareshkumar N (2019). Second law analysis in radiative mixed convective squeezing flow of Casson fluid between parallel disks with Soret and Dufour effects. Heat Transf. Asian Res..

[29] Liu C, Zheng L, Lin P, Pan M, Liu F (2019). Anomalous diffusion in rotating Casson fluid through a porous medium. Phys. A Stat. Mech. Appl..

[30] Abbas Z, Jafar MA, Hasnain J (2020). Analysis of asymptotic solutions for non-Newtonian fluid flow between two parallel discs with dissimilar in-plane motion. Eur. J. Mech.-B/Fluids.

[31] Noranuar WNIN, Mohamad AQ, Shafie S, Khan I, Jiann LY, Ilias MR (2021). Non-coaxial rotation flow of MHD Casson nanofluid carbon nanotubes past a moving disk with porosity effect. Ain Shams Eng. J..

[32] Devaki B, Pai NP, VS SK (2021). Analysis of MHD flow and heat transfer of Casson fluid flow between porous disks. J. Adv. Res. Fluid Mech. Therm. Sci..

[33] Jafar MA, Abbas Z, Hasnain J (2021). Thermally stratified radiative flow of non-Newtonian fluid between two discs executing diverse type of in-plane motion. Case Stud. Therm. Eng..

[34] Akolade MT (2021). Thermophysical impact on the squeezing motion of non-Newtonian fluid with quadratic convection, velocity slip, and convective surface conditions between parallel disks. Partial Differ. Equ. Appl. Math..

[35] Madhukesh, J. K., Ramesh, G. K., Shehzad, S. A., Chapi, S., & Kushalappa, I. P. Thermal transport of MHD Casson–Maxwell nanofluid between two porous disks with Cattaneo–Christov theory. *Numer. Heat Transf. Part A Appl.* **84**, 1–16. 10.1080/10407782.2023.2214322 (2023).

[36] Shehzad SA, Abbasi FM, Hayat T, Alsaedi A (2016). Cattaneo-Christov heat flux model for Darcy–Forchheimer flow of an Oldroyd-B fluid with variable conductivity and non-linear convection. J. Mol. Liq..

[37] Hayat T, Nazar H, Imtiaz M, Alsaedi A (2017). Darcy–Forchheimer flows of copper and silver water nanofluids between two rotating stretchable disks. Appl. Math. Mech..

[38] Ijaz Khan M, Alzahrani F (2020). Entropy optimized magnetohydrodynamics Darcy–Forchheimer second order velocity slip flow of nanomaterials between two stretchable disks. Proc. Inst. Mech. Eng. Part C J. Mech. Eng. Sci..

[39] Riasat S, Ramzan M, Kadry S, Chu YM (2020). Significance of magnetic Reynolds number in a three-dimensional squeezing Darcy–Forchheimer hydromagnetic nanofluid thin-film flow between two rotating disks. Sci. Rep..

[40] Khan MI (2021). Transportation of hybrid nanoparticles in forced convective Darcy–Forchheimer flow by a rotating disk. Int. Commun. Heat Mass Transf..

[41] Siddiqui BK, Batool S, Malik MY, ulhassan QM, Alqahtani AS (2021). Darcy Forchheimer bioconvection flow of Casson nanofluid due to a rotating and stretching disk together with thermal radiation and entropy generation. Case Stud. Therm. Eng..

[42] Shahzad A, Imran M, Tahir M, Khan SA, Akgül A, Abdullaev S, Park C, Zahran HY, Yahia IS (2023). Brownian motion and thermophoretic diffusion impact on Darcy–Forchheimer flow of bioconvective micropolar nanofluid between double disks with Cattaneo-Christov heat flux. Alex. Eng. J..

[43] Basit MA, Farooq U, Imran M, Fatima N, Alhushaybari A, Noreen S, Eldin SM, Akgül A (2023). Comprehensive investigations of (Au-Ag/Blood and Cu-Fe_3_O_4_/Blood) hybrid nanofluid over two rotating disks: Numerical and computational approach. Alex. Eng. J..

[44] Khan SA, Yasmin S, Waqas H, Az-Zo'bi EA, Alhushaybari A, Akgül A, Hassan AM, Imran M (2023). Entropy optimized Ferro-copper/blood based nanofluid flow between double stretchable disks: Application to brain dynamic. Alex. Eng. J..

[45] Cattaneo C (1948). Sulla conduzione del calore. Atti Sem. Mat. Fis. Univ. Modena.

[46] Christov CI (2009). On frame indifferent formulation of the Maxwell-Cattaneo model of finite-speed heat conduction. Mech. Res. Commun..

[47] Hayat T, Qayyum S, Imtiaz M, Alsaedi A (2017). Flow between two stretchable rotating disks with Cattaneo-Christov heat flux model. Results Phys..

[48] Shehzad, S. A., Mushtaq, T., Abbas, Z. & Rauf, A. Double-diffusive Cattaneo–Christov squeezing flow of micropolar fluid. *J. Therm. Anal. Calorim.* **143**, 445–454 (2021).

[49] Bhattacharyya A, Seth GS, Kumar R, Chamkha AJ (2020). Simulation of Cattaneo-Christov heat flux on the flow of single and multi-walled carbon nanotubes between two stretchable coaxial rotating disks. J. Therm. Anal. Calorim..

[50] Tulu A, Ibrahim W (2020). MHD slip flow of CNT-ethylene glycol nanofluid due to a stretchable rotating disk with Cattaneo–Christov heat flux model. Math. Probl. Eng..

[51] Shaw S (2021). Impact of Cattaneo–Christov heat flux on Al_2_O_3_–Cu/H_2_O–(CH_2_OH)_2_ hybrid nanofluid flow between two stretchable rotating disks. Energy Systems and Nanotechnology.

[52] Zeb H, Bhatti S, Khan U, Wahab HA, Munir T, Malik MY (2023). Cattaneo-Christov heat flux modeling in nanofliuid TiO_2_–titanium oxide and aggregation nanoparticle flow between two rotating disks. Waves Random Complex Media.

[53] Noreen S, Farooq U, Waqas H, Fatima N, Alqurashi MS, Imran M, Akgül A, Bariq A (2023). Comparative study of ternary hybrid nanofluids with role of thermal radiation and Cattaneo-Christov heat flux between double rotating disks. Sci. Rep..

[54] Thriveni K, Mahanthesh B (2021). Sensitivity analysis of nonlinear radiated heat transport of hybrid nanoliquid in an annulus subjected to the nonlinear Boussinesq approximation. J. Therm. Anal. Calorim..

[55] Miller R, Griffiths PT, Hussain Z, Garrett SJ (2020). On the stability of a heated rotating-disk boundary layer in a temperature-dependent viscosity fluid. Phys. Fluids.

[56] Turkyilmazoglu M (2016). Flow and heat simultaneously induced by two stretchable rotating disks. Phys. Fluids.

